# Comparative study on nutrient depletion-induced lipidome adaptations in *Staphylococcus haemolyticus* and *Staphylococcus epidermidis*

**DOI:** 10.1038/s41598-018-20801-7

**Published:** 2018-02-05

**Authors:** Yu Luo, Muhammad Afzal Javed, Harry Deneer

**Affiliations:** 1 Department of Biochemistry, College of Medicine, University of Saskatchewan, Saskatoon, Saskatchewan Canada; 2 Department of Microbiology and Immunology, College of Medicine, University of Saskatchewan, Saskatoon, Saskatchewan Canada; 3 Department of Pathology and Laboratory Medicine, College of Medicine, University of Saskatchewan, Saskatoon, Saskatchewan Canada; 4 Molecular Microbiology Laboratory, Division of Clinical Microbiology, Saskatoon Health Region, Saskatoon, Saskatchewan Canada

## Abstract

*Staphylococcus* species are emerging opportunistic pathogens that cause outbreaks of hospital and community-acquired infections. Some of these bacteria such as methicillin-resistant *Staphylococcus* aureus (MRSA) are difficult to treat due to their resistance to multiple antibiotics. We carried out a comparative study on the lipidome adaptations in response to starvation in the two most common coagulase-negative *Staphylococcus* species: a *S. epidermidis* strain sensitive to ampicillin and erythromycin and a *S. haemolyticus* strain resistant to both. The predominant fatty acid composition in glycerolipids was (17:0–15:0) in both bacteria. During the exponential phase, the two bacterial lipidomes were similar. Both were dominated by diacylglycerol (DAG), phosphatidylglycerol (PG), lysyl-phosphatidylglycerol (Lysyl-PG) and Diglucosyl-diacylglycerol (DGDG). Alanyl-PG was detected in small amounts in both bacterial lipids. N-succinyl-lysyl-PG was detected only in *S. haemolyticus,* while lysyl-DAG only in *S. epidermidis*. As the two bacteria entered stationary phase, both lipidomes became essentially nitrogen-free. Both bacteria accumulated large amounts of free fatty acids. Strikingly, the lipidome of *S. epidermidis* became dominated by cardiolipin (CL), while that of *S. haemolyticus* was simplified to DGDG and PG. The *S. epidermidis* strain also produced acyl-phosphatidylglycerol (APG) in the stationary phase.

## Introduction

Most bacteria must live through periods of starvation. The adaptations they have evolved to cope with such a common environmental stress may differ greatly from one another. The bacterial membrane not only acts as the permeation barrier, but also serves as an indicator of metabolic state. Although changes in fatty acid compositions associated with adaptations to extreme temperature and pH^1,2^ have been the focus of studies to date, changes in polar lipid head groups have also been increasingly associated with adaptations to environmental challenges. For instance, bacteria thriving at extremely high pH tend to produce more CL as well as bis-mono-acyl-glycero-phosphate (BMP)^3^. When bacteria enter the stationary phase, activity of cardiolipin synthase has been observed to increase in model organisms *Escherichia coli*^4^ and *Bacillus subtilis*^5^, indicating CL is an essential component in bacterial lipidome when nutrients are depleted. There is also evidence that the lipidome of *B*. *subtilis* becomes dominated by CL in the stationary phase^6^.

Gram-positive *Staphylococcus* strains are an emergent health threat due to their widespread resistance to multiple antibiotics^7,8^. Virulence between species of this genus of bacteria vary. *S. epidermidis* is the most commonly isolated species of coagulase-negative *Staphylococci*, which is an ubiquitous part of the skin flora of humans^9^, and causes the highest numbers of hospital-acquired infections associated with biofilm formation on indwelling medical devices^10,11^. *S*. *haemolyticus*^9^ is also a common part of the skin flora of humans and domestic animals, and the second-most frequently isolated coagulase-negative *Staphylococci*^12^. It is a well-known opportunistic pathogen with the highest level of resistance to multiple antibiotics amongst coagulase-negative *Staphylococci* that forms biofilms and is difficult to treat^12,13^. Lipids extracted from approximately 20 *Staphylococci* species appear to share the major components of PG, CL, DGDG and lysyl-PG, as well as the absence of phosphatidylethanolamine (PE)^14^. It would be interesting to discover differences in their lipidome adaptations to cope with environmental stress, which may shed light on their differences in long-term survival on medical devices.

Many Gram-positive bacteria are known to produce glycolipids^15^ as well as aminoacylated lipids, especially lysyl-PG^16^. Biosynthesis of such lipids is catalyzed by multiple peptide resistance factor (MprF) which is also known as lysyl-phosphatidylglycerol synthase^17,18^. Aminoacylated lipids harbor a positively charged ammonium group, and are important for resistance to cationic host immune peptide such as defensins^19^. The crystal structures of two MprF enzymes have been determined, which may serve as the targets for novel antibiotics^20^. We have recently employed TLC and MS in profiling of aminoacylated phospholipids in the Gram-positive model organism of *B*. *subtilis*^21^. An ampicillin-resistant bacterium that produced N-succinyl-lysyl-phosphatidylglycerol^22^ was identified as *S*. *haemolyticus*, a species that drew immediate attention to other *Staphylococci* pathogens. Despite apparent similarities in their lipidome composition in the exponential phase, *S*. *epidermidis* and *S*. *haemolyticus* appeared to have evolved with drastically different lipidome adaptations in the stationary phase. *S*. *epidermidis* appeared to switch to an “usual” CL-dominated lipidome and also produced APG in the stationary phase, while the lipidome of *S*. *haemolyticus* appeared to simplify to essentially only two components of DGDG and PG. Due to nutrient depletion, both bacteria ceased to produce aminoacylated phospholipids while accumulating large amounts of free fatty acids with 12 to 22 carbons, which implies normal anabolic activity of fatty acid biosynthesis but severe inhibition of lipid biosynthesis in the stationary phase as demonstrated in glycerol-starved *E*. *coli*^23^ and *B*. *subtilis*^24^.

## Results

### Profiling and tandem mass spectrometry of lipid extract from *S. epidermidis* and *S. haemolyticus* in exponential growth phase

**Figure 1:**
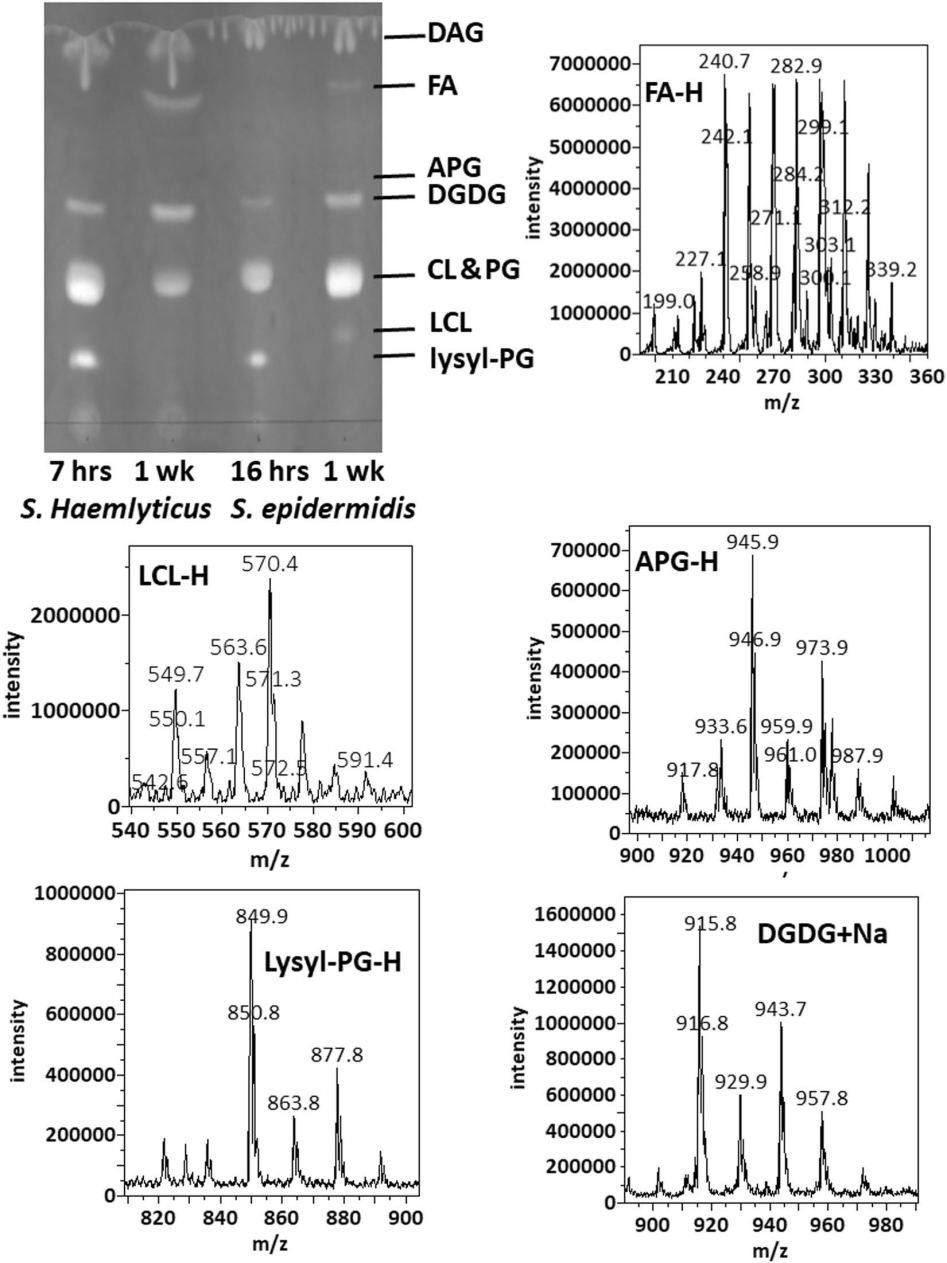
Thin-layer chromatogram and mass spectra of lipids extracted from *S. epidermidis* and *S. haemolyticus*. The TLC sheet was stained with 0.02% primuline. Representative fluorescent bands were analyzed by MS. The lipid compositions are marked on the right. DAG was partially recovered from the solvent front. Mass spectra in recovered lipids from representative TLC bands are shown. CL migrated slightly faster than PG, but not enough to separate the two types of lipids by TLC.

**Figure 2:**
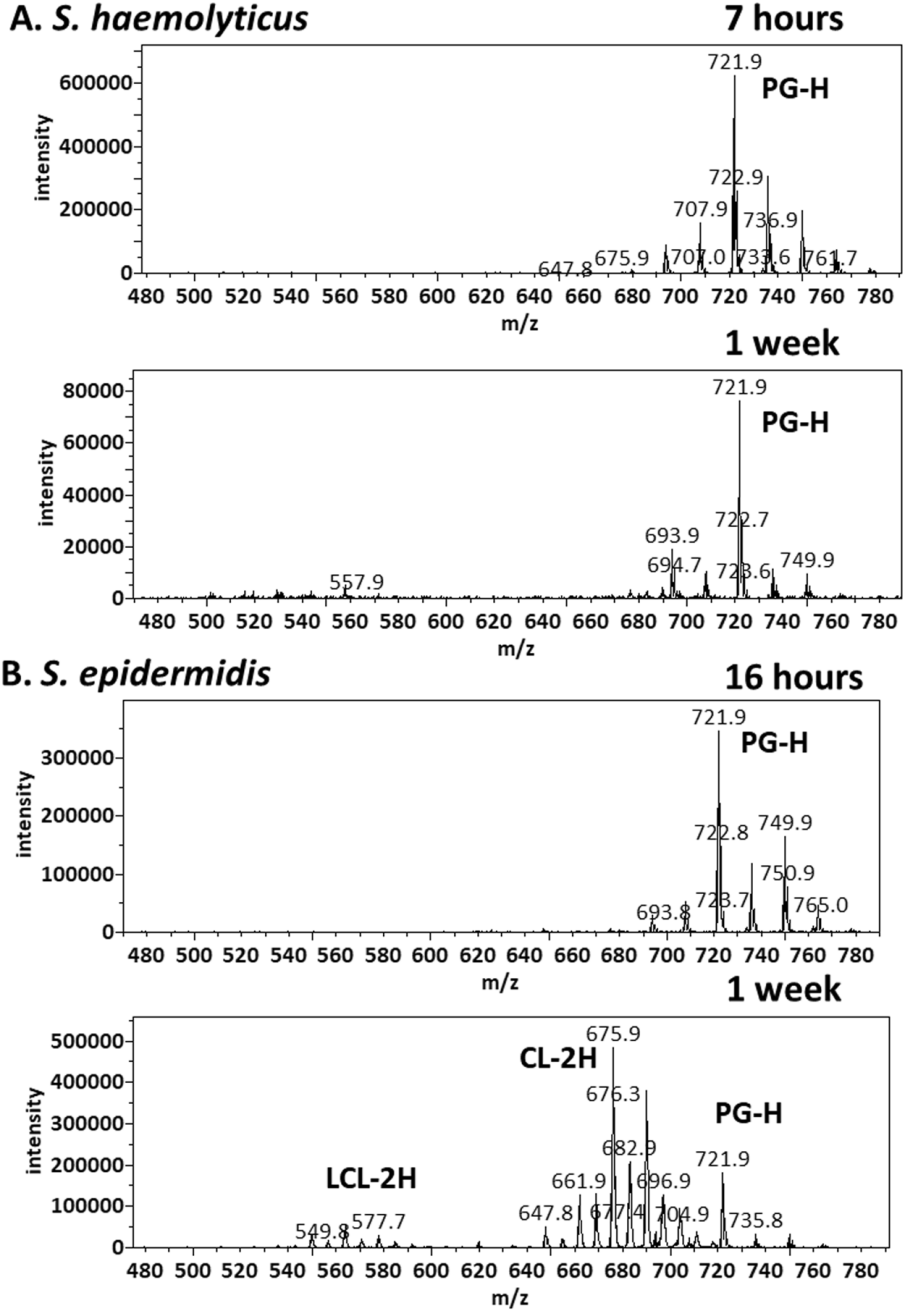
Reduced level of PG during stationary phase and production of CL and LCL in *S. epidermidis* during stationary phase. Precursor scans for 153 m/z anionic head group fragment are shown. The most abundant peaks corresponding to PG anions (721 m/z), CL double anions (675 m/z) and LCL double anions (549 m/z) are labeled.

**Figure 3:**
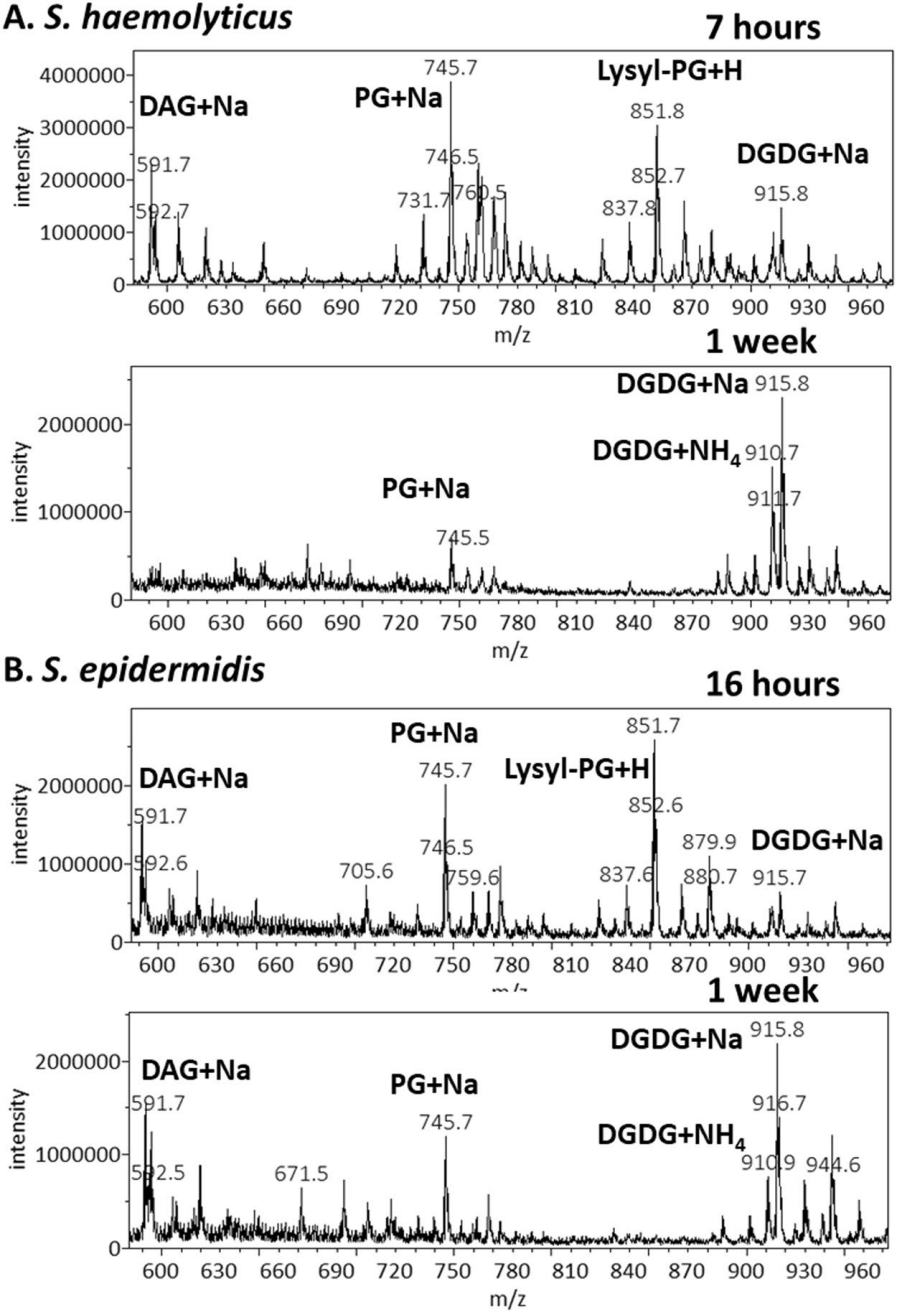
Absence of lysyl-PG during stationary phase. Positive MS spectra are shown. The most abundant peaks corresponding to DAG, PG, lysyl-PG and DGDG cations are labeled. PG was observed as a cluster of sodiated ions centered at 745 m/z. A cluster of protonated lysyl-PG ions was observed around 851 m/z. More sodiated DGDG ions (around 915 m/z) were observed than ammoniated DGDG (around 910 m/z). Sodiated DAG cations (around 591 m/z) were also observed.

We cultured *S. epidermidis* ad *S. haemolyticus* in LB media. *S. haemolyticus* grew faster and reached a maximum optical density of 2.0 after 15 hours. *S. epidermidis* was slower, reaching a maximum optical density of 1.9 after 36 hours. Replicates of *S. haemolyticus* cultures in the exponential phase were harvested 7 hours after inoculation. They had an optical density of 1.5 and pH of 7.0. *S. epidermidis* cultures were harvested 16 hours after inoculation, with an observed optical density of 1.3 and pH of 7.0. Lipids were extracted using the most common Bligh-Dyer method^25^ but with all solvents chilled on ice^26^. Three replicates of each of the two sets of lipid extracts were profiled by both MS and TLC (Fig. 1). In addition to polar lipids, we also observed abundance of ammoniated and sodiated cations of the non-polar lipid of DAG in the positive mass spectrum (supplementary data), which was recovered from silica gels at the solvent front on the TLC sheet (Fig. 1). The MS/MS spectra of these DAG cations were consistent with known patterns^27^ and were manually analyzed to assign fatty acid compositions (supplementary data). As demonstrated in a previous survey of lipids from over ten Staphylococci species, lipid compositions of the two bacteria were similar^14^. Both negative mass spectra were dominated by deprotonated PG ions, followed by those of lysyl-PG, and chloride adduct ions of DGDG. Alanyl-PG anions were barely above noise level in the MS spectra, but were positively identified by a precursor scan for 88 m/z deprotonated alanine (supplementary data). Small amounts of lysyl-DAG were detected in *S. epidermidis* lipids by a neutral loss scan for 146 amu lysine in positive mode (supplementary data). *S. haemolyticus*, however, produced N-succinyl-lysyl-PG as described previously^22^. In both lipidomes, PG was the predominant species followed by lysyl-PG, DGDG, and DAG (Fig. 1). A survey of fatty acid compositions was carried out by manually analyzing tandem mass spectra of the most abundant PG, acyl-PG and lysyl-PG anions as well as sodiated and ammoniated DAG and DGDG cations based on general dissociation patterns known for glycerophospholipids^28^ (supplementary data). The predominant fatty acid composition was found to be (17:0) at the sn-1 position and (15:0) at the sn-2 position, which corresponds to the predominant ions of deprotonated PG at 721 m/z, deprotonated lysyl-PG at 821 mz and double deprotonated CL at 675 m/z (Figs 1 and 2), as well as sodiated PG at 745 m/z and protonated lysyl-PG at 851 m/z (Fig. 3). Predominant DGDG of this fatty acyl composition was observed as sodiated ion at 915 m/z and as ammoniated ion at 910 m/z (Figs 1 and 3), while DAG with this fatty acyl composition was observed as sodiated ion at 591 m/z (Fig. 3) and ammonium ion at 586 m/z.

### Onset of stationary phase-triggered lipidome adaptation

Most bacteria must face undulations in nutrient supply, making starvation more of a norm rather than an exception. We initially analyzed the bacterial lipid extracts at multiple time points between 1 and 7 days. While the transition was noticeable at intermediate time points, cells harvested 1 week after inoculation demonstrated the furthest shift of lipidome composition. Density of *S*. *epidermidis* culture in LB reached a maximum of 1.9 at the end of day 2 before gradual decreasing. One-week-old culture of *S*. *epidermidis* in LB had a residual optical density of 0.4 and pH of 9.0. Biofilm formation was noticeable on the flask wall which may contributed to the decline of cell density in the liquid medium. LB culture of *S. haemolyticus* reached a stable density plateau of 2.0 at the end of day 1. Cell density remained nearly constant afterwards, but pH drifted to 9.0 after 1 week.

**Figure 4:**
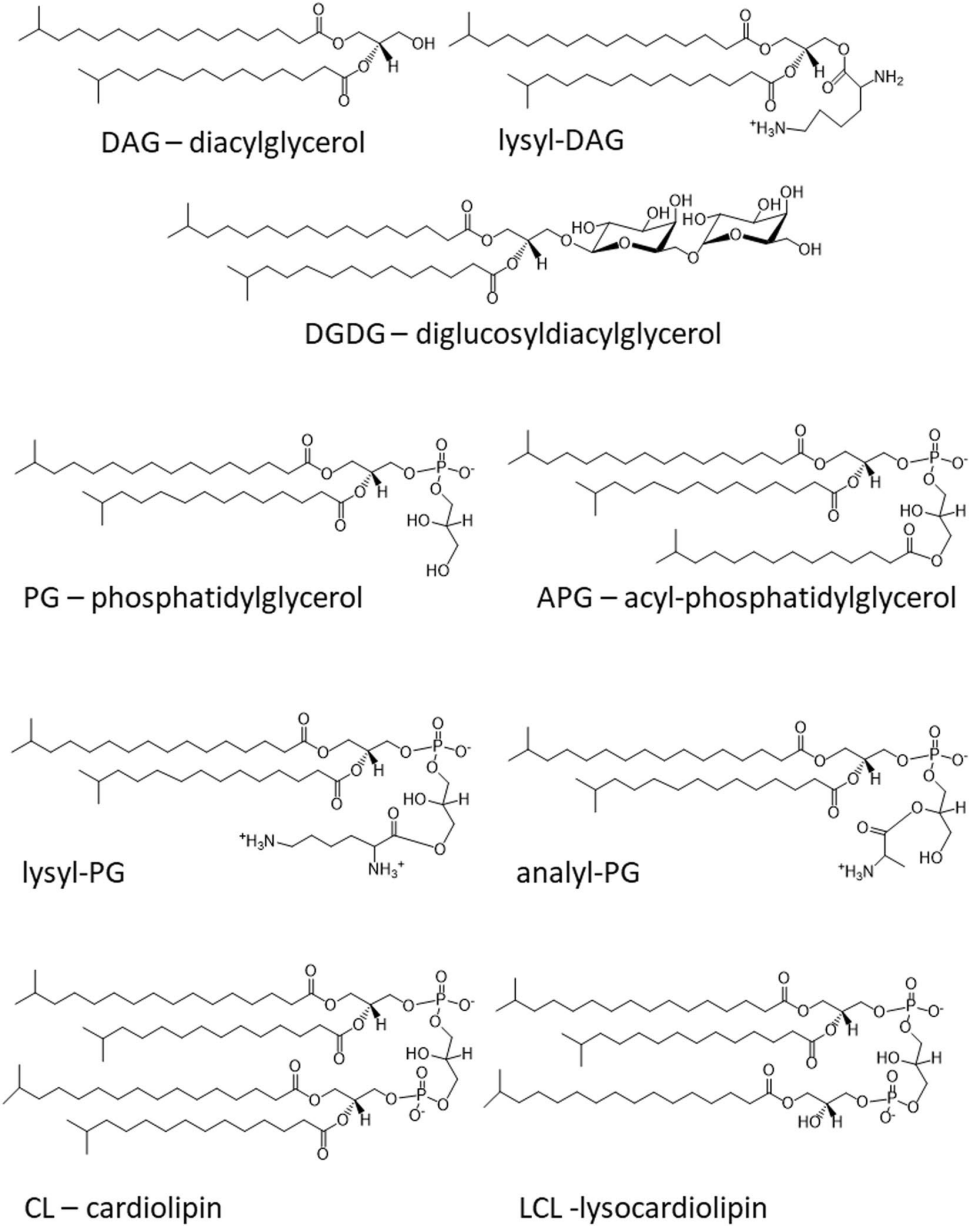
Summary of chemical structures of representative lipids. Chemical structures of DAG, lysyl-DAG, DGDG, PG, APG, lysyl-PG, alanyl-PG, CL and LCL are shown. The predominant (17:0–15:0) fatty acyl composition is shown in these structures. Only iso-branched fatty acyl chains are shown.

A lipid species was noticed only in the lipid extracted from *S*. *epidermidis* in the stationary or cell death phase as a minor fast-migrating compound on the TLC sheet (Fig. 1). It was identified as APG (Fig. 4) by tandem mass spectrometry (supplementary data), which matched the dissociation pattern that has been observed for a chemically synthesized reference compound^29^ and those observed for glycerophospholipids^28^. The third fatty acyl group in all detected APG anions appeared to have a common composition of (15:0).

**Figure 5:**
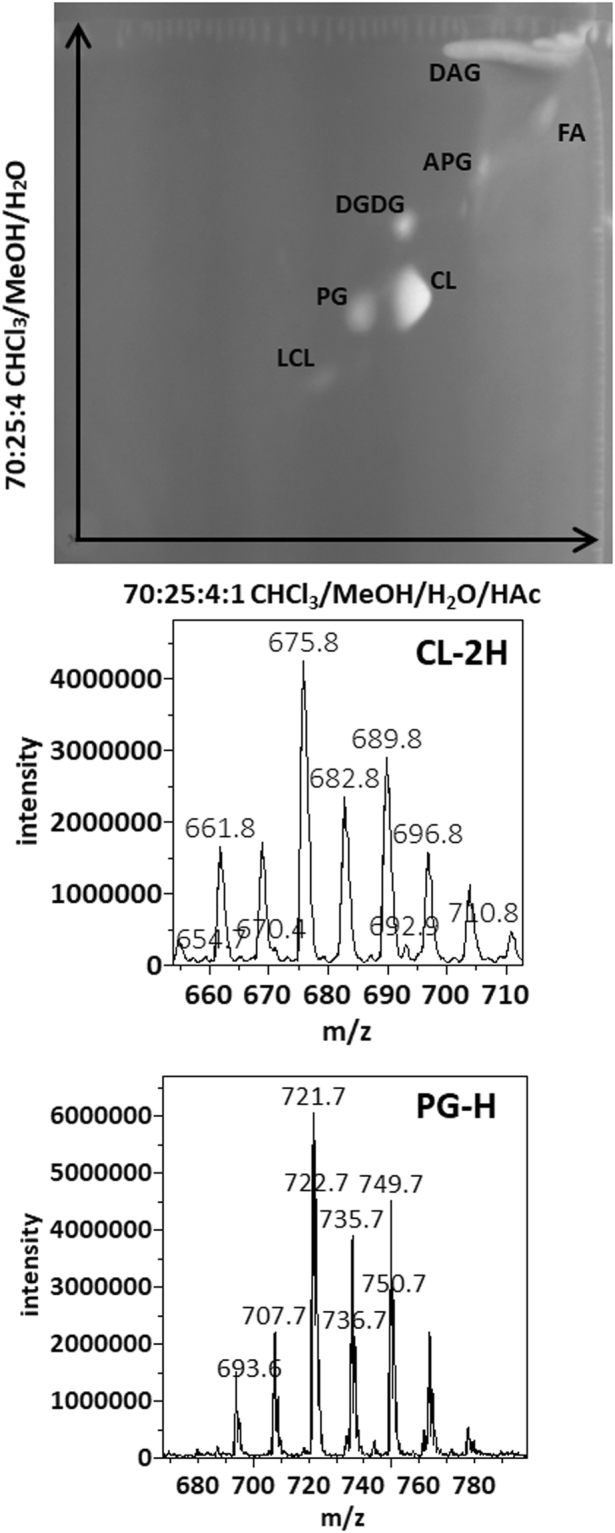
2-dimentional thin-layer chromatogram and mass spectra of lipids extracted from *S. epidermidis* in the stationary phase. The 2D-TLC sheet was stained with 0.02% primuline. Lipids recovered from the fluorescent spots were analyzed by MS. The lipid compositions are marked on the chromatogram. Negative MS spectra of lipids recovered from silica gels at the PG and CL spots are shown.

As shown in (Figs 1–3), TLC analysis and MS profiling in negative and positive modes clearly revealed the differences in lipidome adaptation in the two coagulase-negative *Staphylococcus* species. Lipid extracts from both bacteria in the stationary phase were observed to contain a rather large composition of free fatty acid (FA), which was visible as the two most intense white band while the TLC sheet was till wet with primuline spray but not particularly intense in fluorescence (Fig. 1). On the other hand, all aminoacylated lipids were undetected in these lipid extracts (Figs 1 and 3). They were undetected even with the more sensitive method of precursor scans for 88 m/z alanine anion and 145 m/z lysine anion. Production of APG in *S*. *epidermidis* appeared to be growth phase-dependent (Fig. 1). CL migrated slightly faster than PG, resulting in overlap of the two types of lipids on 1D-TLC chromatogram developed with the mobile phase of 70:25:4 chloroform/methanol/water (Fig. 1). It’s worth noting only lipids extracted from *S*. *epidermidis* in the stationary phase appeared to have noticeable amount of CL and LCL. We analyzed the *S*. *epidermidis* lipid extract by 2D-TLC with the following two mobile phases: 70:25:4 chloroform/methanol/water and 70:25:4:1 chloroform/methanol/water /acetic acid. The acidic mobile phase successfully separated PG from CL and showed that the fluorescent spot corresponding to CL was by far the brightest on the 2D-TLC chromatogram (Fig. 5). Therefore, the most noticeable lipidome adaptation in *S*. *epidermidis* was the meteoric rise of CL as the predominant lipid (Figs 1, 2 and 5) accompanied by less dramatic rise of lyso-cardiolipin (LCL) and moderate increase of DGDG, as well as drastic decrease of PG. In *S*. *haemolyticus*, starvation-triggered lipidome adaptation appeared to be extremely different from that observed for *S*. *epidermidis*. CL and LCL were either undetected or detected in trace amounts by precursor scans for precursor anions that generate phosphate or phosphoglycerol fragment in all lipid extracts of *S*. *haemolyticus* (Figs 1 and 2). As this bacterium entered stationary phase, there was a noticeable decrease of PG, a moderate increase of DGDG, and decrease of DAG (Figs 1 and 3). Its lipidome became drastically simplified to only two major components of PG and DGDG.

## Discussion

### Free fatty acids

In the model organisms *E*. *coli*^23^ and *B*. *subtilis*^24^, fatty acid synthesis has been observed to continue even during periods when lipid biosynthesis is inhibited, which leads to accumulation of free fatty acids. Large amounts of saturated fatty acids of 12 to 22 carbon atoms were detected by negative mass spectrum (Fig. 1) in lipids extracted from both *S*. *epidermidis* and *S*. *haemolyticus* in the stationary phase, implying lipid biosynthesis is inhibited to a great extend in both bacteria when challenged with nutrient depletion. This is expected as both bacteria appeared to experience growth arrest or even cell death as indicated by their respective optical density. Cells alive after one week of culture are likely those that have been slowed or arrested in cell division, which no longer need lipid biosynthesis. Accumulation of free fatty acids may also serve as an indication of anabolic activity in these persistent cells. The free fatty acids, however, likely exist in droplets separated from the bacterial membrane. Alternatively, accumulation of free fatty acids may be due to degradation of membrane lipids^30^ as fatty acid biosynthesis is generally inhibited during stationary phase^31^.

### DAG and DGDG

DGDG is produced as a membrane lipid as well as the lipid anchor of lipoteichoic acids in Gram-positive bacteria^32^. DAG can accumulate in the membrane as the phosphoglycerol head group of PG is transferred to form polymeric lipoteichoic acid^32^. In theory, increase of DGDG in both bacteria could alleviate the consumption of phosphorous resource. Decrease of DAG in *S*. *haemolyticus* implies that lipoteichoic acid biosynthesis in this bacterium may become inhibited during stationary phase.

### Aminoacylated lipids

Absence of PE and abundance of lysyl-PG have been known features in many *Staphylococcus* species^14^. Species-specific production of lysyl-DAG in *S*. *epidermidis*, and that of N-succinyl-lysyl-PG in *S*. *haemolyticus* may be due to widespread mobile genetic elements in *Staphylococci* species^33–35^. Total absence of aminoacylated lipids makes the lipidomes in both bacteria devoid of nitrogen-containing lipids in the stationary phase, which may benefit other essential needs such as protein biosynthesis for long-term survival. This may also represent a general tendency to simplify the lipidome as nutrients become scarce. However, growth phase-dependent synthesis of APG in *S*. *epidermidis* would not fit in this scheme of lipidome simplification. We previously observed that alanylated lipids in *B*. *subtilis* is predominantly D-alanylated^21^ independent of MprF which is the L-lysyl-PG synthase^19^. We speculate that the dltABCD proteins involved in D-alanylation of teichoic acids may be responsible for D-alanylated lipids. Expression of MprF and dltABCD proteins are dependent on Aps antimicrobial-sensing system in Gram-positive bacteria such as *S*. *epidermidis*^36^. Importantly, expression of these proteins is turned off during stationary phase due to the interplay of the Aps system and quorum-sensing Agr system in *S*. *aureus*^37^. During stationary phase, bacteria do not have to face the challenge of antimicrobial peptides, cease of production of aminoacylated lipids would be beneficial to the preservation of amino acids for long-term survival. When bacteria breach the skin, they are greeted by not just abundance of nutrient but also a hostile mix of antimicrobial peptides. As the fresh nutrient triggers bacterial transition to exponential phase, enhanced antimicrobial peptides-resistance associated with aminoacylated lipids becomes concomitant essentiality.

### Glycerophospholipids

Accumulation of CL and/or LCL is a relatively well-known strategy observed in starved model organisms *Escherichia coli*^4^ and *Bacillus subtilis*^5^, and in *Acinetobacter* species^38^. In this regard, *S*. *epidermidis* appears to adopt a rather common strategy to cope with nutrient depletion. *S*. *haemolyticus*, on the other hand, appears to have evolved with a completely different strategy in building an extremely simple lipidome essentially made of only DGDG and PG. Surprisingly, this strain of *S*. *haemolyticus* did not appear to produce noticeable amount of CL, a predominant lipid species in many *Staphylococci*^14^.

### Difference in starvation-triggered lipidome adaptation

Surprisingly, the two *Staphylococcus* species showed apparently different strategies in lipidome adaptation at the onset of stationary phase, an indication metabolic pathways are impacted by nutrient depletion differently in these two closely related bacteria. Bacteria thrive in a wide range of biological niches. Hardly any bacterium, *E*. *coli* and *B*. *subtilis* included, can be considered an ideal model organism, no matter how much has already been learned from it. A wide survey will be needed to gain a more complete knowledge of bacterial lipidome adaptations in response to environmental stress factors. Slow-growing or growth-arrested bacteria tend to be much more tolerant to antibiotics^39^. Study of bacteria in stationary phase may shed light on how bacteria manage to survive under harsh environments. They may adopt different lipidome while growth is slowed or arrested. Understanding these differences may help innovate lipidome composition-specific antimicrobial agents to combat emerging antibiotic-resistant pathogens.

## Methods

### Bacterial strain and cell culture

The strain of *S. haemolyticus* was previously isolated in the lab as an ampicillin-resistant bacterium^22^. This organism was identified by matrix-assisted laser desorption ionization-time of flight (MALDI-TOF) mass spectrometry using the bioMerieux Vitek MS system in the Clinical Microbiology Laboratory, Saskatoon Health Region. *S. epidermidis* was a skin isolate obtained from the Culture Collection in the Department of Microbiology and Immunology. Bacterial cells were cultured overnight in autoclaved Luria-Bertani (LB) liquid medium from freezer stocks. All cell incubation was carried out in an environmental shaker at 37 °C and 220 RPM. The overnight cultures were diluted 1000-fold into 50 ml LB media and then incubated for specified durations. After measuring pH with pH paper and cell density at 600 nm, the cell suspension was transferred to a 50-ml centrifuge tube and mixed with 0.75 ml 1.0 M Sodium acetate buffer at pH 5.2. Centrifugation was carried out at 4,000 rpm for 16 minutes at 4 °C using a Beckman-Coulter TS-5.1–500 rotor in an Allegra 25 centrifuge. The wet cell pellet was rinsed twice with ice-chilled ddH2O. The pellet was resuspended by aspiration in a volume of 1:4 mixed solvent of ddH2O/methanol in proportion to the optical density of the bacterial culture. Typically, bacterial cell pellet from 50 ml of LB culture harvested at an optical density of 1.5 was resuspended in 2.5 mL mixed solvent for lipid extraction.

### Lipid extraction

HPLC-grade organic solvents (Fisher Scientific, Ottawa, ON, Canada) and distilled deionized water were used throughout the experiments. Bacterial lipids were extracted following the Bligh-and-Dyer method^25^ with glassware and ice-chilled solvents as previously described^26^. After adding chloroform and then water to the cell suspension in ddH_2_O/methanol for lipid extraction and eventual phase separation, centrifugation at 1,300 rpm for 5 minutes was carried out to enhance phase separation. The bottom chloroform-rich phase was transferred to a second tube and mixed with 0.5 ml 0.5 M NaCl. After shaking by hand for 1 minute, phase separation was again enhanced by centrifugation. The final chloroform-rich phase, approximately 4.5 ml for a typical starting point of 2.5 ml cell suspension in ddH_2_O/methanol, was transferred to a third tube for storage at −80 °C.

### Lipid profiling by Mass Spectrometry

Each lipid sample was mixed in a syringe with two-fold volume of methanol supplemented with 5 mM NH_4_OH prior to direct infusion at a rate of 0.9 ml/hr. MS spectra and precursor scans were acquired with a QTRAP 4000 LC-MS/MS (Applied Biosystems, Foster City, CA, USA) mass spectrometer equipped with a Turbo V Ion Spray electrospray ionization (ESI) source. Optimal electrospray ionization was achieved at a temperature of 400 °C and ionization voltage of -4500 V or +5500 V for negative and positive modes, respectively. The precursor scans were acquired with collision energies set at −65 or +45 eV in negative or positive mode, respectively. The SCIEX Analyst 1.6 software was used to acquire and export averaged mass spectra. Mass spectra were also inspected by Mass++ 2.7.4 software^40^. The MS figures were generated by Mass++ 2.7.4. Chemical structures were drawn using PerkinElmer ChemDraw prime 15.101.144.

### Tandem mass spectrometry

MS/MS spectra were also acquired using the QTRAP 4000 mass spectrometer in the Enhanced-Product-Ion mode. Energy spread was fixed at 10 eV. Collision energy was optimized in the range of 30 to 70 eV. Typical optimized collision energies were 65 eV and 45 eV in negative and positive modes, respectively.

### Lipid analysis by thin-layer chromatography

500 µl of lipid sample was dried in vacuum at an ambient temperature of 21 °C for 10 minutes, and resuspended in 30 µl 4:1 chloroform/methanol. A glass capillary tube was used to spot the resuspension onto an EMD Millipore TLC plastic sheet cut to a height of 10 cm. After drying in air at the ambient temperature for 20 minutes, the TLC sheet was placed in a TLC chamber pre-equilibrated with a mixed solvent of chloroform/methanol/water (70:25:4). After 25 minutes of development, the TLC sheet was removed from the TLC chamber and dried for 20 minutes at the ambient temperature. The TLC sheet was stained with 0.02% primuline (Sigma-Aldrich) solution in 80:20 acetone/water, and then dried in air at the ambient temperature for an hour. The fluorescent image was recorded with a Syngene G:BOX system. The silica gels at each representative fluorescent band were scraped with a metal spatula and transferred to an Agilent glass sample vial. 200 µl of chloroform was added before storing at −20 °C. 400 µl methanol was added to the silica gel suspension right before MS analysis. After shaking by hand for 10 seconds followed by sedimentation of silica gels for at least 2 minutes, the clarified sample was subjected to MS analysis by direct infusion. MS scan, precursor scan, neutral loss scan, and MS/MS spectra were acquired to assign lipid composition in each TLC fluorescent band.

## Electronic supplementary material

Supplementary data
