# Supplementary material for: Comparative study on nutrient depletion-induced lipidome adaptations in Staphylococcus haemolyticus and Staphylococcus epidermidis
Source: Sci Rep. 2018 Feb 5;8:2356. doi: 10.1038/s41598-018-20801-7 (PMC5799182; doi:10.1038/s41598-018-20801-7)
Supplement: Supplementary file 1 — Supplementary data [file 41598_2018_20801_MOESM1_ESM.pdf]

Comparative study on nutrient depletion-induced lipidome adaptations in *Staphylococcus haemolyticus* and *Staphylococcus epidermidis*

Yu Luo[*1], Muhammad Afzal Javed[2], Harry Deneer[3,4]

[1] Department of Biochemistry, College of Medicine, University of Saskatchewan, Saskatoon, Saskatchewan, Canada

[2] Department of Microbiology and Immunology, College of Medicine, University of Saskatchewan, Saskatoon, Saskatchewan, Canada

[3] Department of Pathology and Laboratory Medicine, College of Medicine, University of Saskatchewan, Saskatoon, Saskatchewan, Canada

[4] Molecular Microbiology Laboratory, Division of Clinical Microbiology, Saskatoon Health Region, Saskatoon, Saskatchewan, Canada

*To whom correspondence should be addressed: Prof. Yu Luo, Telephone: 1 306 966-4379; Fax: 1 306 966-4390; Email: yu.luo@usask.ca

Supplementary data

Introduction: Here we present the microbial typing results, and the manually assigned fatty acid compositions based on tandem mass spectrometry acquired with the 4000 QTRAP system. The fatty acyl assignment was mainly based on dissociation patterns described in the following two articles:

Murphy, R.C. and P.H. Axelsen, *Mass spectrometric analysis of long-chain lipids.* Mass Spectrom Rev, 2011. **30**(4): p. 579-99.

Coulon, D. and Bure, C. Acylphosphatidylglycerol (acyl-PG) or N-acylphosphatidylethanolamine (NAPE). J. Mass Spectrom, 2015. 50. 1318-20

In negative mode, the $[FA-H]^-$ ion dissociated from the sn-2 position is generally 2-3 fold more abundant than that from the sn-1 position. We did a rather complete survey of PG and lysyl-PG. The compositions of DAG and DGDG were based on the assumption they were produced from the same pool of DAG for synthesizing phospholipids. Their MS/MS spectra in positive mode, especially their $[DAG-OH]^+$ and $[MAG-OH]^+$ fragments, were used to confirm the two fatty acyl composition.

Abbreviations: L – lyso, as in LPA, LPG and LCL; FA – fatty acid; MAG – monoacylglycerol; DAG – diacylglycerol; DGDG – diglucosyldiacylglycerol; PG – phosphatidylglycerol; APG – acyl-phosphatidylglycerol; PE – phosphatidylethanolamine; PA – phosphatic acid; CL – cardiolipin.

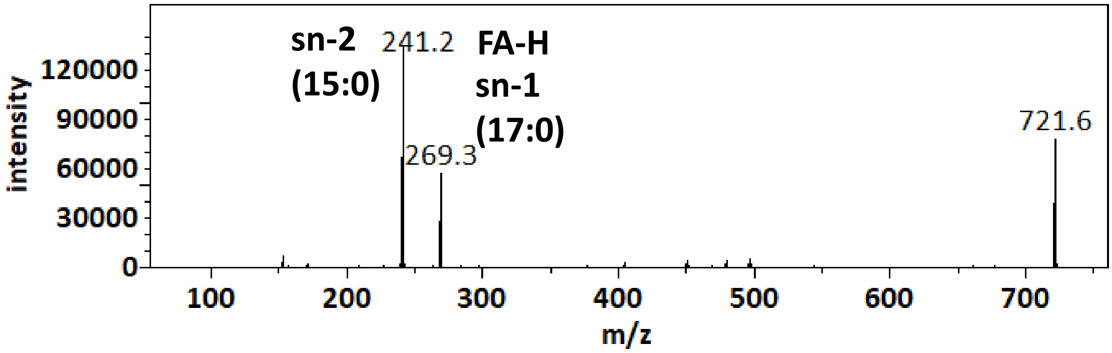

Figure S1. MS/MS spectrum of 721 m/z [PG-H]$^-$ ion.

Since PG anions were the most abundant, we were able to acquire meaningful MS/MS spectra of several of such anions and assigned their fatty acid compositions, as listed in table S1. These compositions serve as a near complete survey of fatty acid compositions in glycerophospholipids found in the two *staphylococcus* strains. Many of these PG anions were mixtures of multiple fatty acid compositions, which are listed in the order of descending abundance of corresponding FA anions in the MS/MS spectra. As we follow the general rule that fatty acid anion dissociated from the sn-2 position is more abundant by 2-3 fold, it appeared that fatty acyls at the sn-1 position were always equal to or larger that those at the sn-2 position. The minor compositions of FA pairs were assigned based on their equal overall mass to that of the major composition and that the ion counts of each pair of FA anions differ by 2-3 fold.

**Table S1. [PG - H]$^-$ (m/z)**	**fatty acid composition**
679	15:0-14:0, 16:0-13.0
693	15:0-15:0, 16:0-14:0
707	16:0-15:0, 17:0-14:0, 18:0-13:0
**721**	**17:0-15:0**, 18:0-14:0
735	18:0-15:0, 19:0-14:0
749	19:0-15:0, 20:0-14:0, 18:0-16:0, 17:0-17:0
763	20:0-15:0, 18:0-17:0, 19:0-16:0

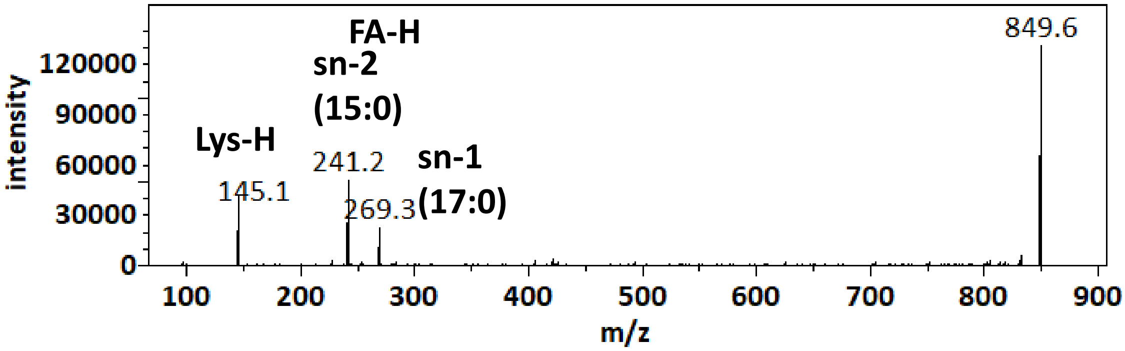

Figure S2. MS/MS spectrum of 849 m/z [lysyl-PG-H]$^-$ ion.

Since lysyl-PG anions were also abundant, we were able to acquire meaningful MS/MS spectra of several of such anions and assigned their fatty acid compositions, as listed in table S2. As in PG anions, it appeared that fatty acyls at the sn-1 position were always equal to or larger that those at the sn-2 position.

**Table S2. [lysyl-PG - H]$^-$ (m/z)**	**fatty acid composition**
821	15:0-15:0
835	16:0-15:0
**849**	**17:0-15:0**
863	18:0-15:0
877	19:0-15:0
891	20:0-15:0, 18:0-17:0

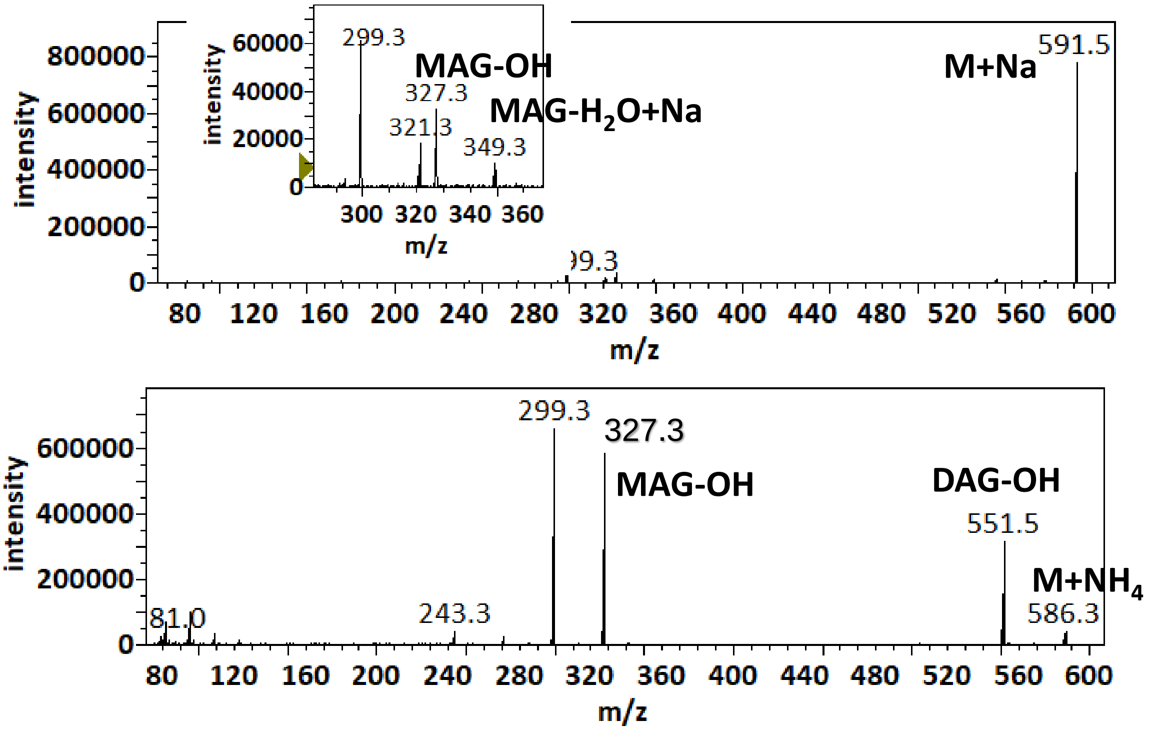

Figure S3. MS/MS spectra of 591 m/z and 586 m/z DAG cations

Most DAG ions observed were sodiated. It dissociated into [MAG-OH]$^+$ and [MAG-H_2O+Na]$^+$ ions separated by 22 m/z. We acquired MS/MS spectrum of the most abundant 591 m/z [DAG+Na]$^+$ ion. We also acquired tandem MS spectra of several [DAG+NH_4]$^+$ ions, which produced strong signals of [DAG-OH]$^+$ as well as [MAG-OH]$^+$ fragment ions. We also assumed the sn-1 position is occupied by the larger fatty acyl group, as suggested by MS/MS spectra of PG and lysyl-PG anions. The fatty acyl compositions are listed in table S3.

Table S3.

**[DAG + Na]$^+$ (m/z)**	**fatty acid composition**
**591**	**17:0-15:0**
**[DAG + NH_4]$^+$ (m/z)**	**fatty acid composition**
558	15:0-15:0
572	16:0-15:0, 17:0-14:0
**586**	**17:0-15:0**
600	18:0-15:0
614	19:0-15:0, 17:0-17:0

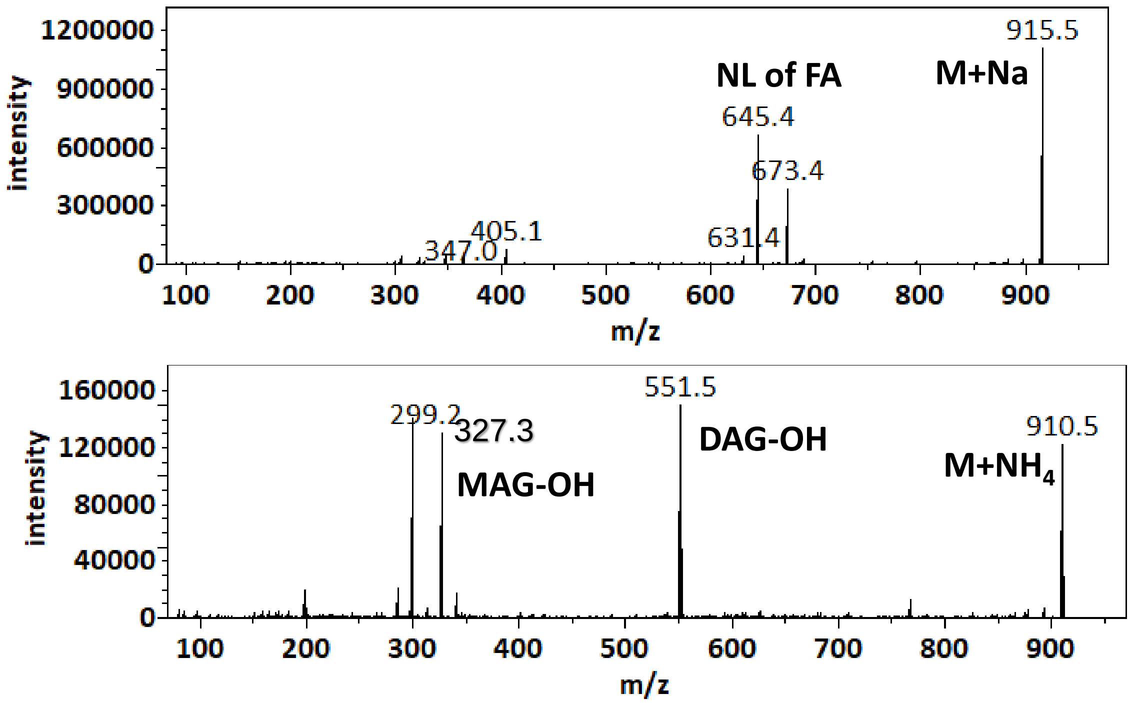

Figure S4. MS/MS spectra of 915 m/z and 910 m/z DGDG cations

DGDG anions were not abundant in negative mode, and they overlap with lysyl-PG anions with larger fatty acyl groups. Sodiated DGDG ion s were chosen as the target species to survey this type of lipids by mass spectrometry. These cations produced strong signals due to neutral loss of FAs, which were used to confirm the identity of the two fatty acyl groups. We also acquired tandem MS spectra of several $[DGDG+NH_4]^+$ ions, which produced extremely strong signals of $[DAG-OH]^+$ as well as $[MAG-OH]^+$ fragment ions. The assigned fatty acyl compositions are listed in table S4.

Table S4.

**$[DGDG + Na]^+$ (m/z)**	**fatty acid composition**
915	17:0-15:0
929	18:0-15:0
943	19:0-15:0, 17:0-17:0
**$[DGDG + NH_4]^+$ (m/z)**	**fatty acid composition**
910	17:0-15:0
924	18:0-15:0
938	19:0-15:0, 17:0-17:0

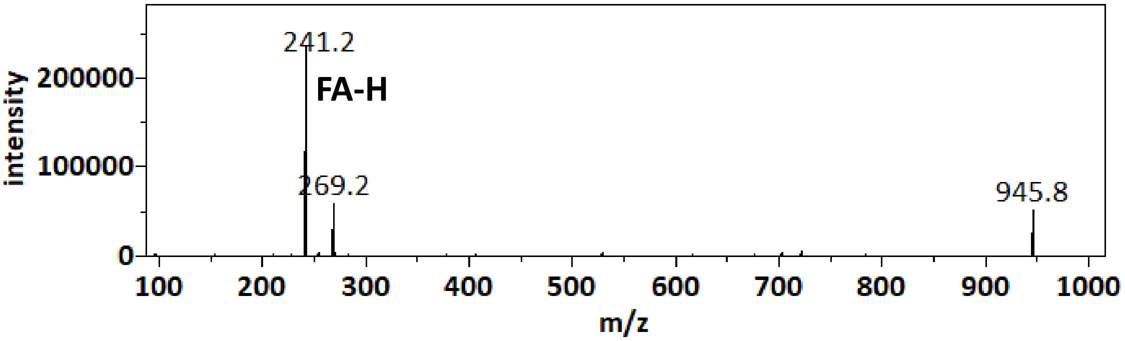

Figure S5. MS/MS spectrum of 945 m/z [APG-H]$^-$ ion.

Tri-acylated lipid APG was only observed in lipids extracted from *S. epidermidis* in the stationary phase. After purification by TLC, we were able to acquire meaningful MS/MS spectra of four such anions and assigned their fatty acid compositions, as listed in table S5. The two fatty acyl compositions of the DAG moiety and that of the head group glycerol is separated by a slash. We noticed that all these four MS/MS spectra shared one feature that the 241 m/z fragment ion was at least 4-fold as intense as other fatty acid anions. This intensity difference could not be explained by the difference at the sn-1 and sn-2 positions in PG or lysyl-PG, which is normally 2.5 fold favoring the sn-2 fatty acid anion. We tentatively interpreted this unusual intensity of the 241 anion as suggesting that all APG is likely formed by acylation of the PG head group by a (15:0) fatty acid (242 amu). In addition to the strong signals at 241 m/z and 269 m/z corresponding to (15:0) and (17:0) FA anions, this MS/MS spectrum also revealed much weaker signals of (14:0) and (18:0) FA anions at 227 m/z and 283 m/z, respectively. Remarkably, intensity ratio of the two weaker signals was 2.2, indicating a (18:0-14:0) minor composition of the phosphatidyl group, and leading to the assignment of a common (15:0) fatty acyl to the second glycerol moiety.

**Table S5. [APG - H]$^-$ (m/z)**	**fatty acid composition**
917	15:0-15:0 / 15:0
**945**	**17:0-15:0 / 15:0**, 18:0-14:0 / 15:0
959	18:0-15:0 / 15:0, 17:0-16:0 / 15:0
973	19:0-15:0 / 15:0, 17:0-17:0 / 15:0

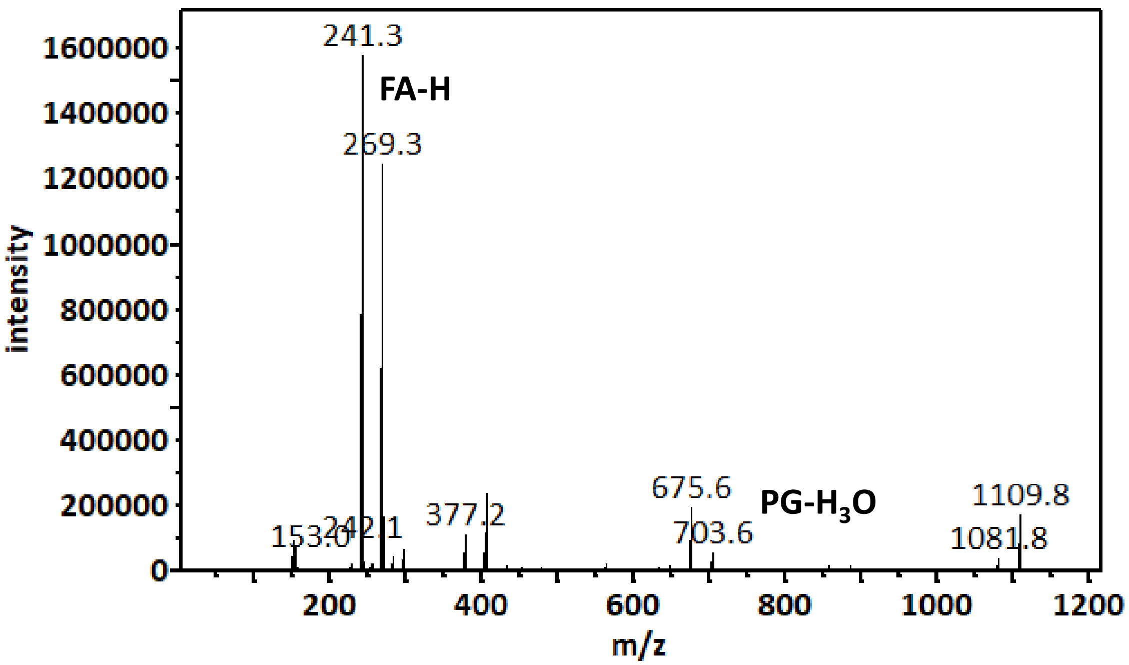

Figure S6. MS/MS spectrum of 675 m/z $[CL\text{-}2H]^{2-}$ ion.

Both CL and LCL were observed in noticeable amounts only in lipids extracted from *S. epidermidis* in the stationary phase. After purification by TLC, we were able to acquire meaningful MS/MS spectra of several CL double anions and assigned their fatty acid compositions, as listed in table S6. The two fatty acyl compositions of the two phosphatidyl moieties were based on FA anions and cyclic PG anions. For instance, the above spectrum suggested a cyclic PG anion at 703 m/z, matching a fatty acid composition of (32:0). The FA anions at 241 and 269 m/z suggest the fatty acids are (17:0) and (15:0) at the sn-1 and sn-2 positions, respectively.

There was a rather wide spectrum of CL produced by S. epidermidis during the stationary phase. Their fatty acid compositions were assigned manually and listed in Table S6.

**Table S6. [CL - 2H]$^{2-}$ (m/z)**	**fatty acid composition**
640	15:0-15:0 / 15:0-14:0
647	15:0-15:0 / 15:0-15:0
654	16:0-15:0 / 15:0-15:0
661	17:0-15:0 / 15:0-15:0
668	17:0-15:0 / 16:0-15:0
**675**	**17:0-15:0 / 17:0-15:0**
682	17:0-15:0 / 18:0-15:0
689	17:0-15:0 / 19:0-15:0
696	17:0-15:0 / 20:0 -15:0, 18:0-15:0 / 19:0-15:0
703	19:0-15:0 / 19:0-15:0,
710	19:0-15:0 / 20:0-15:0

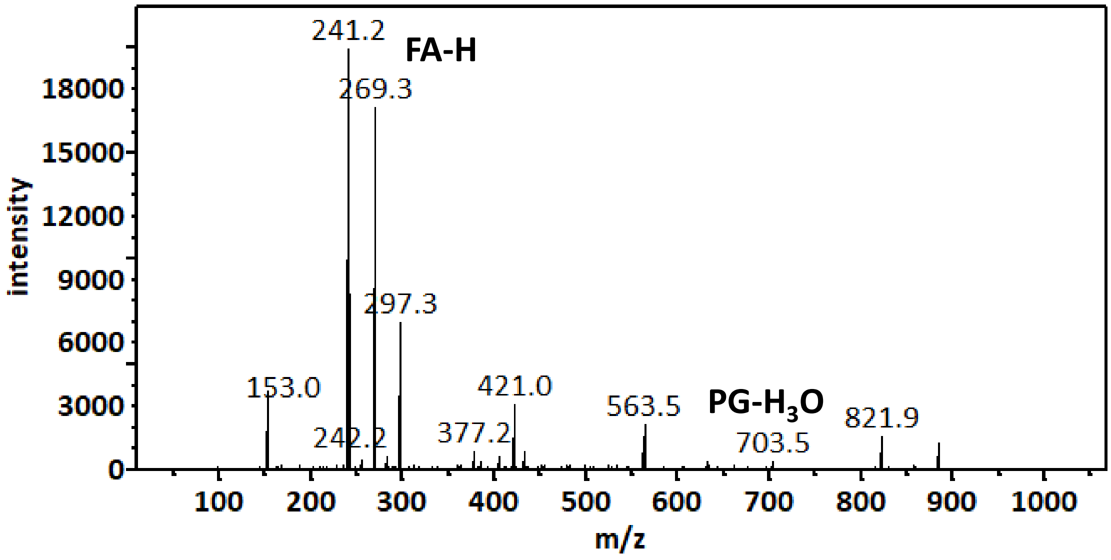

Figure S7. MS/MS spectrum of 563 m/z [LCL-2H]$^{2-}$ ion.

Both CL and LCL were observed in noticeable amounts only in lipids extracted from *S. epidermidis* in the stationary phase. After purification by TLC, we were able to acquire meaningful MS/MS spectra of several LCL double anions and assigned their fatty acid compositions, as listed in table S7. The two fatty acyl compositions of the two phosphatidyl moieties were based on FA anions and cyclic PG anions. For instance, the above spectrum suggested a cyclic PG anion at 703 m/z, matching a fatty acid composition of (32:0). The FA anions at 241 and 269 m/z suggest the fatty acids are (17:0) and (15:0) at the sn-1 and sn-2 positions, respectively. An extra (17:0) FA must be assigned to the other glycerol moiety.

There was much less LCL than CL anions that were observed at adequate intensity for MS/MS analysis. The longer FA on the sn-1 position of the mono-acylated glycerol moiety appeared to be preserved in LCL, implying activity of a phospholipase A2.

Table S7. [LCL - 2H]$^{2-}$ (m/z)	fatty acid composition
**5**49	17:0-15:0 / 15:0
**563**	**17:0-15:0 / 17:0**
570	18:0-15:0 / 17:0
577	19:0-15:0 / 17:0
584	20:0-15:0 / 17:0
591	19:0-15:0 / 19:0

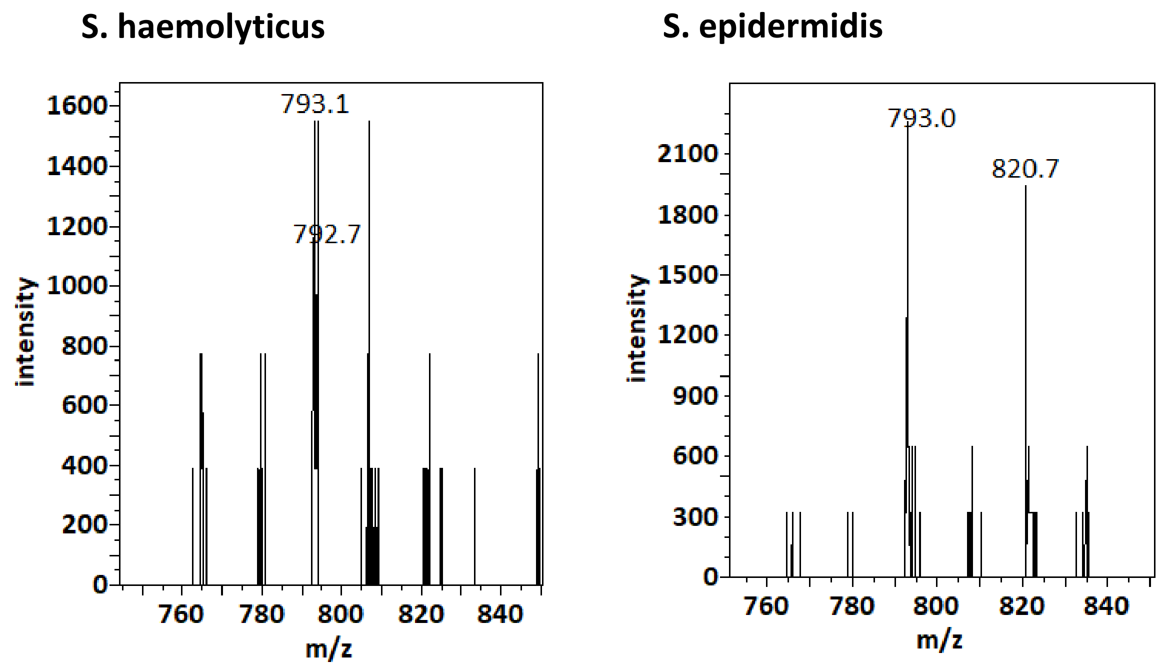

Figure S8. Precursor scan for 88 m/z [Alanine-H]⁻ fragment.

Alanyl-PG was detected in lipids extracted from both *S. epidermidis* and *S. haemolyticus* in the exponential growth phase. They were in low quantities and did not form a visible band in thin-layer chromatogram. The 792 m/z anion corresponds to alanyl-PG with a fatty acid composition of (17:0-15:0).

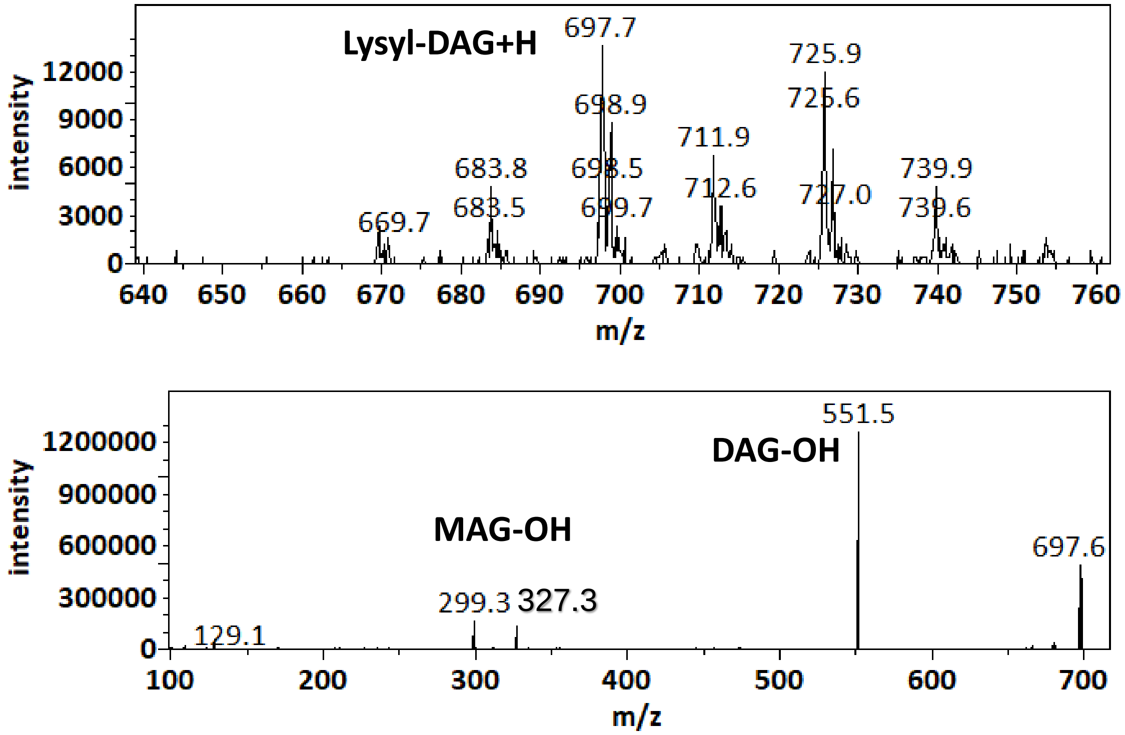

Figure S9. Top -MS scan in positive mode for neutral loss of 146 amu lysin. Bottom – MS/MS spectrum of the 697 m/z cation.

Lysyl-DAG was observed in lipids extracted from *S. epidermidis* in the exponential phase. It did not form a visible band on thin-layer chromatogram. We could not rule out the possibility that this lipid species may be the product of spontaneous chemical reaction between two major lipids of DAG and lysyl-PG in the extract.

The MS/MS spectrum of the dominant lysyl-DAG cation is shown. As in MS/MS spectra of ammoniated DAG and DGDG ions, strong signals corresponding to $[MAG\text{-}OH]^+$ and $[DAG\text{-}OH]^+$ fragment ions of expected m/z values were observed. The 551 m/z fragment corresponds to the neutral loss of lysine (146 amu) which would produce a $[DAG\text{-}OH]^+$ ion with a fatty acyl composition of (17:0-15:0). $[MAG\text{-}OH]^+$ ions at 299 and 327 m/z would be consistent with the two fatty acyls in the dehydroxylated DAG cation. Although we could not rule out isobaric glutamyl-DAG, the double positive charge in lysyl-PG would make it a biologically more efficient molecule to introduce positive charge.

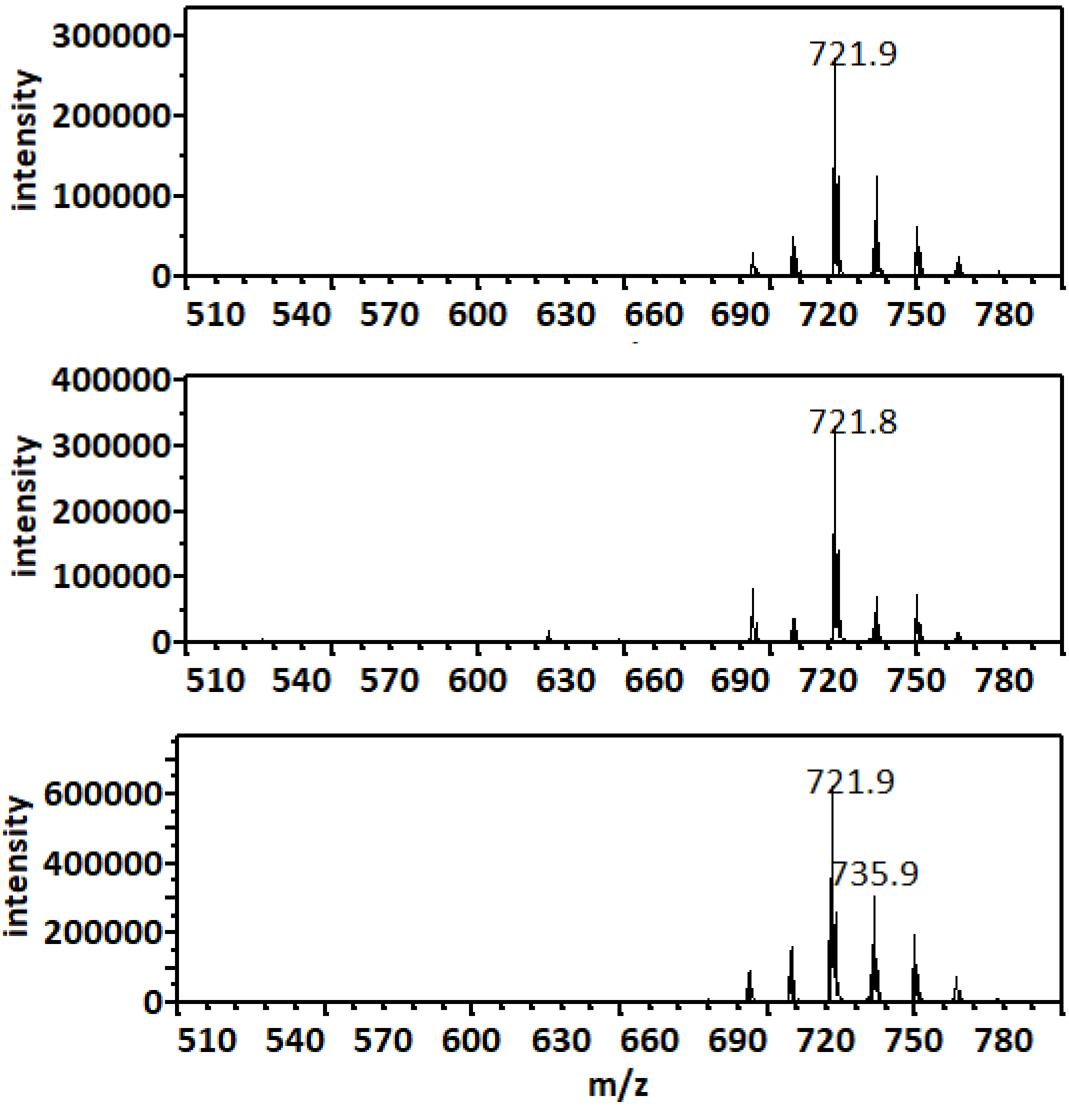

Figure S10. Precursor scans for -153 m/z cyclo-phosphoglycerol fragment in 3 lipid samples of *S. haemolyticus* in exponential phase.

The peaks were clustered arounf 721 m/z separated by 14 m/z, indicating lack of cardiolipin or lysocardiolipin double anions which would be separated by 7 m/z. Cardiolipin double anions was expected to cluster around a major peak at 675 m/z, which was not observed. As shown in Figure S1 and Table S1, lack of any monolyso- or dilyso-cardiolipin fragments in the MS/MS spectra of the major precursors further indicates that they are PG anions with varied fatty acid composition.

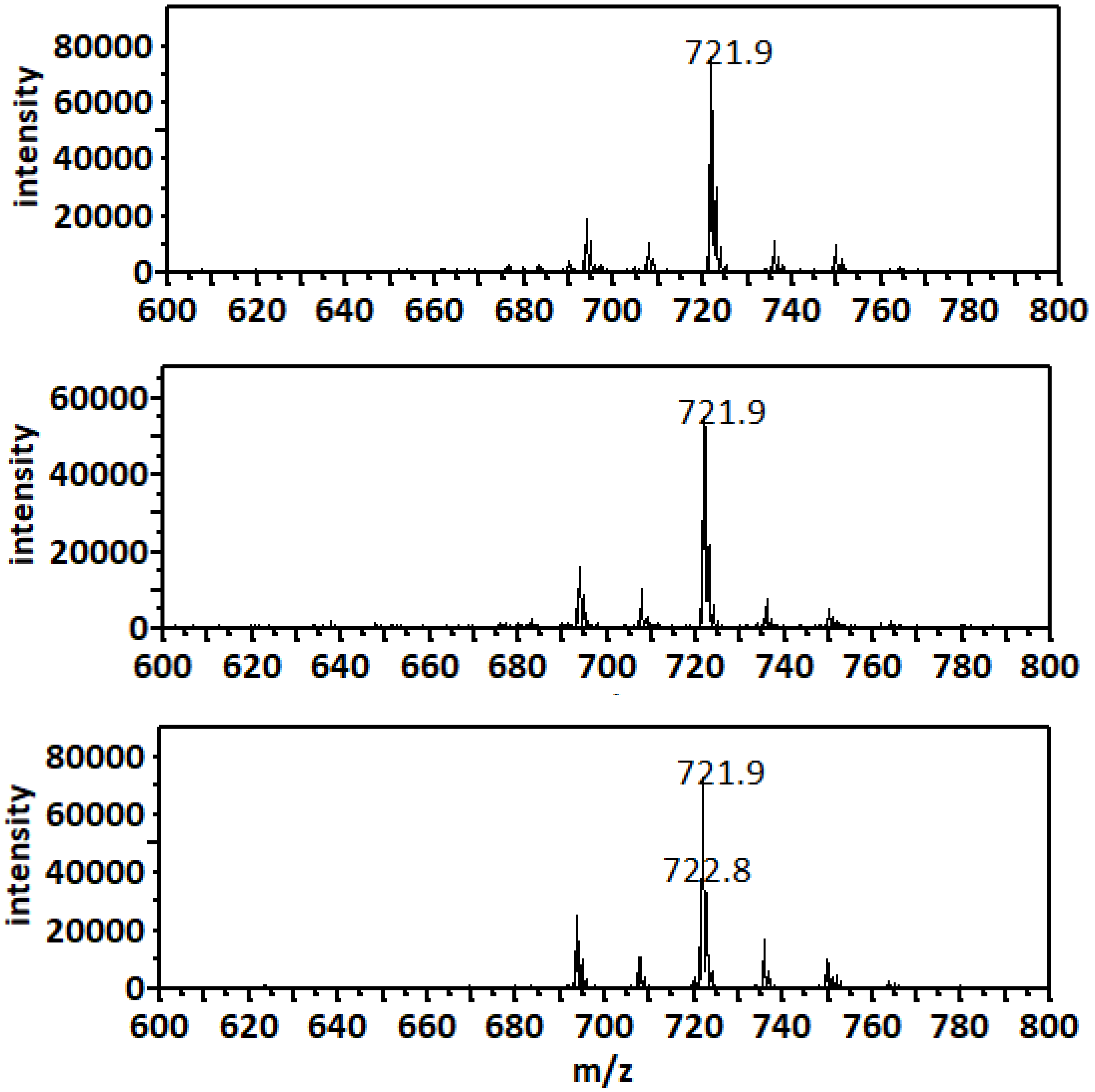

Figure S11. Precursor scans for -153 m/z cyclo-phosphoglycerol fragment in 3 lipid samples of *S. haemolyticus* in stationary phase.

In comparison with the precursor scans of lipids from *S. haemolyticus* in exponential phase shown in Figure S10, the profile of the spectra shown above are very similar except for noticeable decrease in intensity by nearly an order of magnitude. There was no indication cardiolipin or lysocardiolipin accumulated in the bacterial membrane during stationary phase. Collectively, our data suggests that *S. haemolyticus* produces less PG as it enters stationary phase.

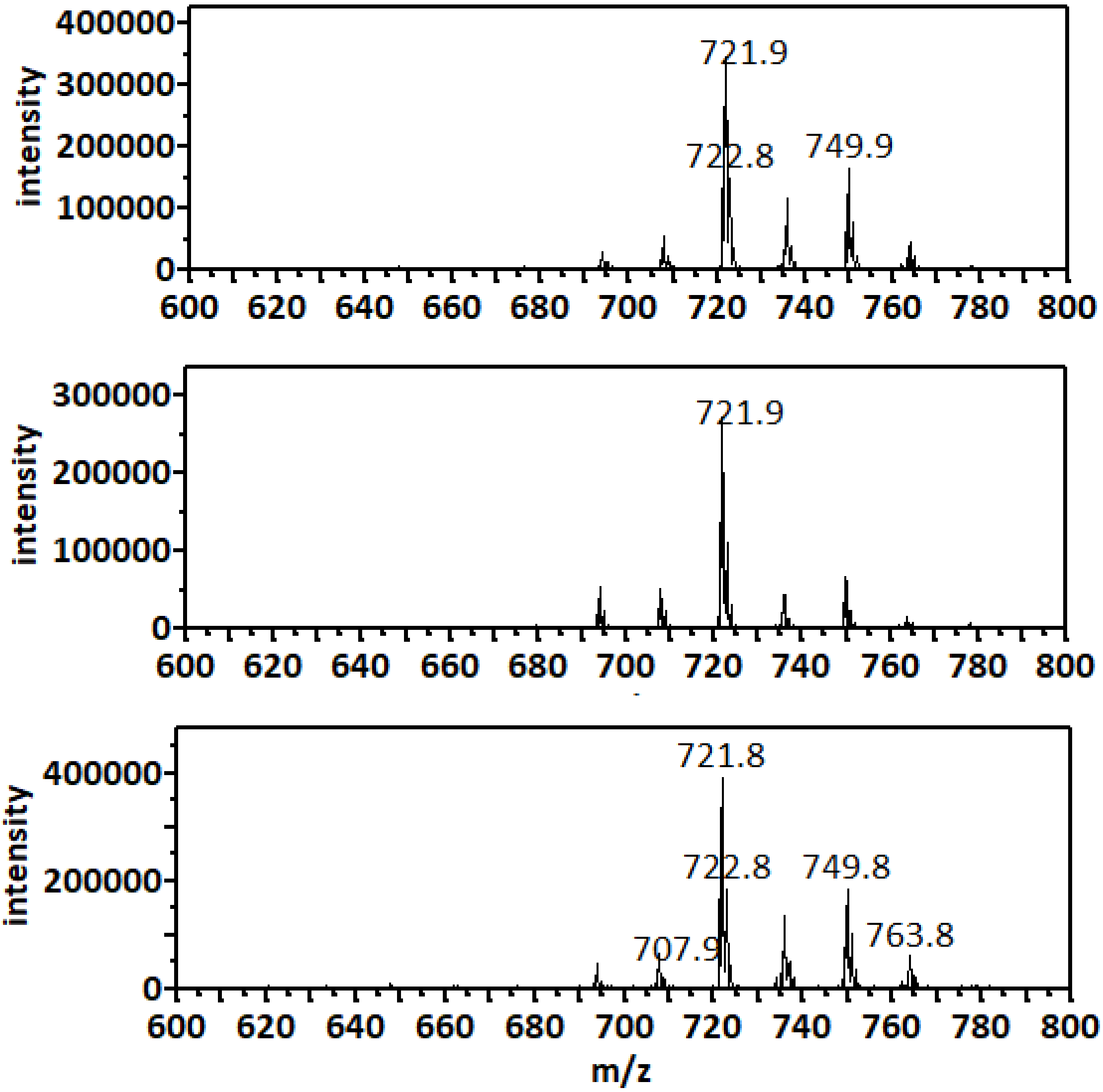

Figure S12. Precursor scans for -153 m/z cyclo-phosphoglycerol fragment in 3 lipid samples of *S. epidermidis* in exponential phase.

The peaks were clustered around 721 m/z separated by 14 m/z, indicating lack of cardiolipin or lysocardiolipin double anions which would be separated by 7 m/z. Cardiolipin double anions was expected to cluster around a major peak at 675 m/z, which was not observed. As shown in Figure S1 and Table S1, lack of any monolyso- or dilyso-cardiolipin fragments in the MS/MS spectra of the major precursors further indicates that they are PG anions with varied fatty acid composition.

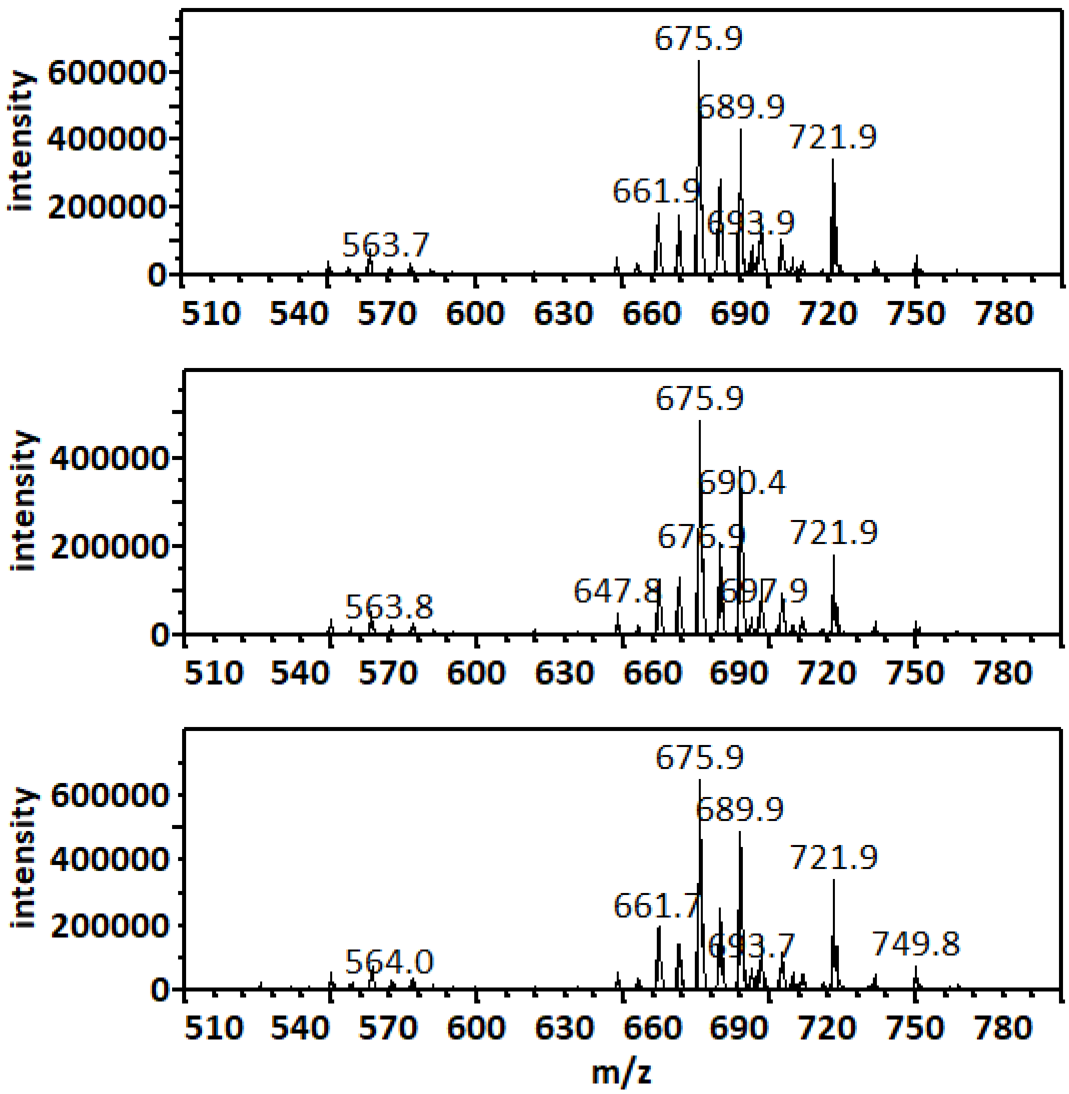

Figure S13. Precursor scans for -153 m/z cyclo-phosphoglycerol fragment in 3 lipid samples of *S. epidermidis* in stationary phase.

In addition to the PG anion cluster around 721 m/z with adjacent peaks separated by 14 m/z, two other clusters emerged with adjacent peaks separated by 7 m/z. As interpreted in Figure S6 and S7 as well as in Table S6 and S7, the cluster around 675 m/z corresponds to cardiolipin double anions, while the cluster around 563 m/z corresponds to lysocardiolipin double anions.

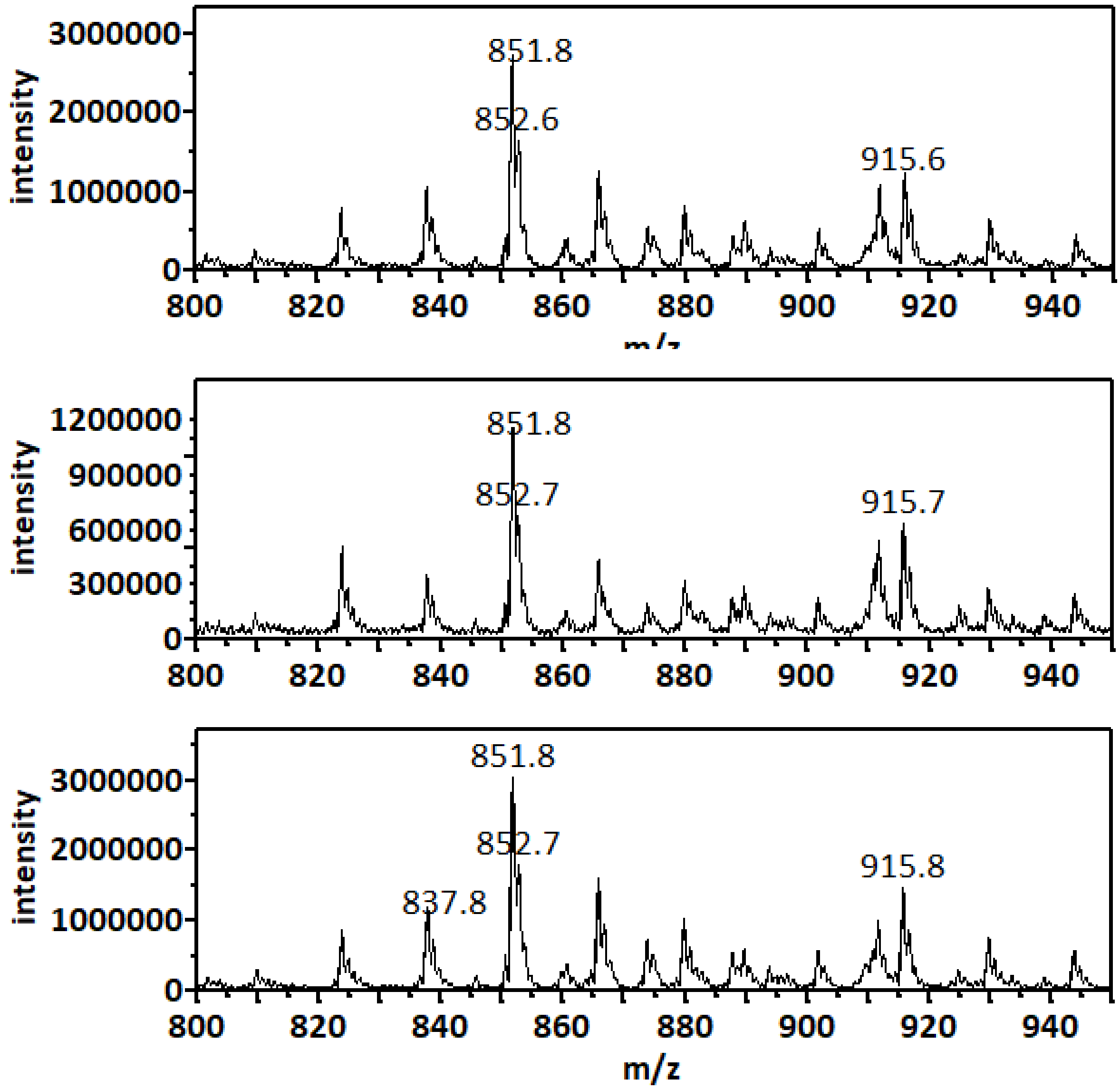

Figure S14. Positive MS spectra of 3 lipid samples of *S. haemolyticus* in exponential phase.

The major peaks were clustered around 851 m/z separated by 14 m/z. They correspond to protonated lysyl-PG cations. Less intense peaks around 915 m/z correspond sodiated DGDG cations, while those around 910 m/z correspond to ammoniated DGDG cations. Both type of lipids were abundant in lipid samples extracted from *S. haemolyticus* in exponential phase.

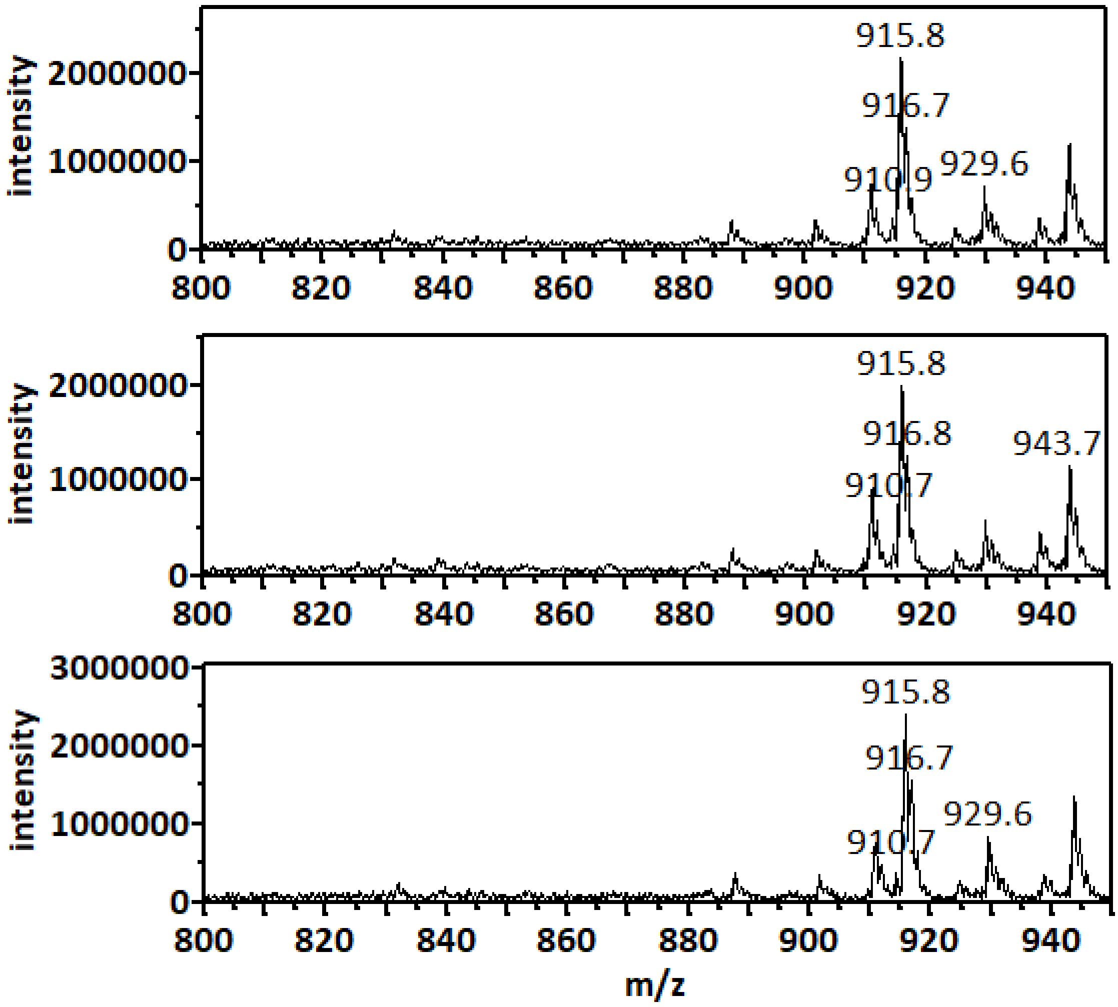

Figure S15. Positive MS spectra of 3 lipid samples of *S. haemolyticus* in stationary phase.

In comparison with MS spectra of lipids from *S. haemolyticus* in exponential phase, two clusters of DGDG ions centered around 910 (ammoniated) and 915 (sodiated) m/z were noticeably more abundant. Protonated Lysyl-PG ions were absent. We also confirmed that deprotonated lysyl-PG ions were also absent using a precursor scan for -145 m/z lysine anion.

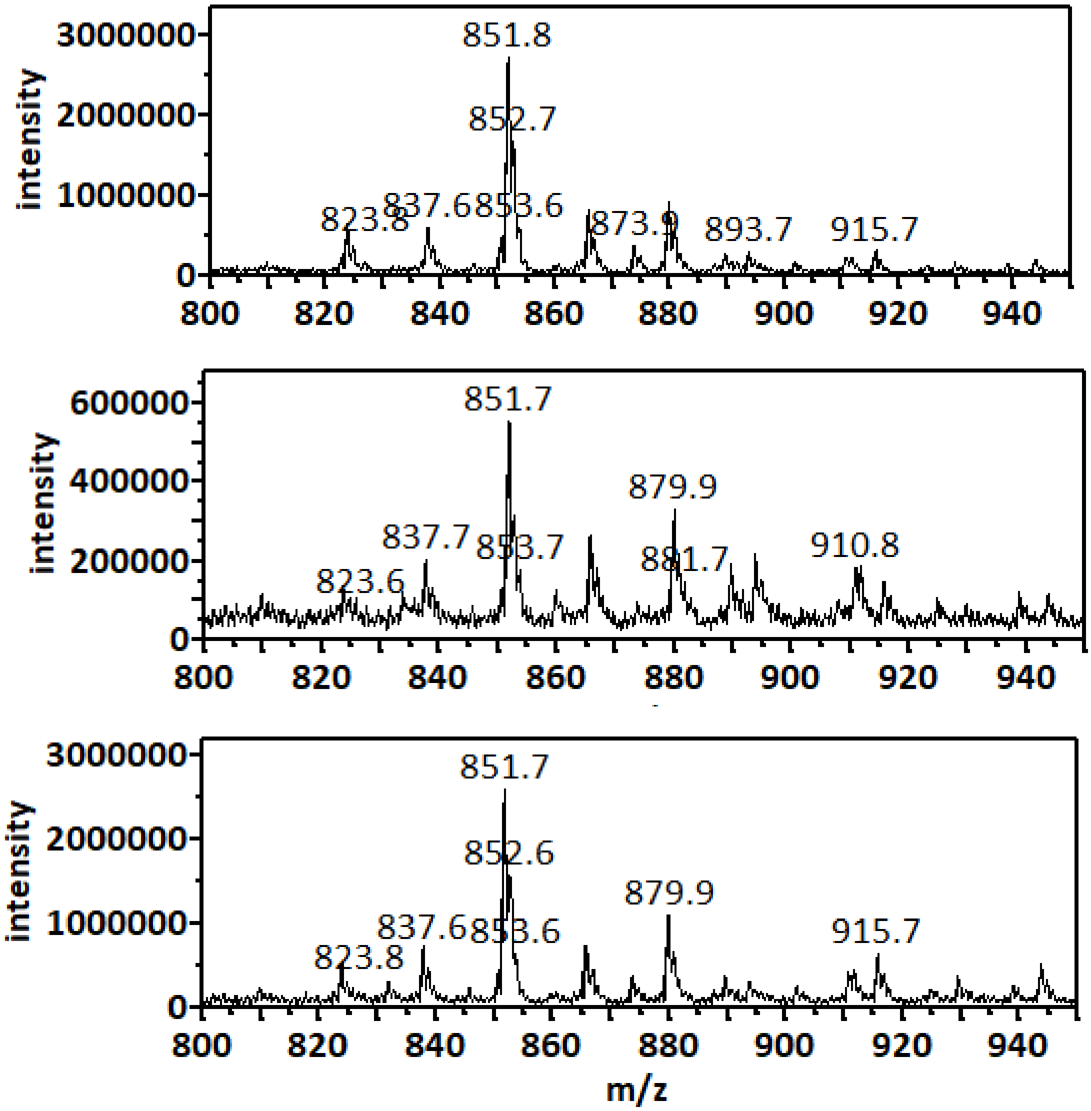

Figure S16. Positive MS spectra of 3 lipid samples of *S. epidermidis* in exponential phase.

The major peaks were clustered around 851 m/z separated by 14 m/z. They correspond to protonated lysyl-PG cations. Less intense peaks around 915 m/z correspond sodiated DGDG cations, while those around 910 m/z correspond to ammoniated DGDG cations. Both type of lipids were abundant in lipid samples extracted from *S. epidermidis* in exponential phase.

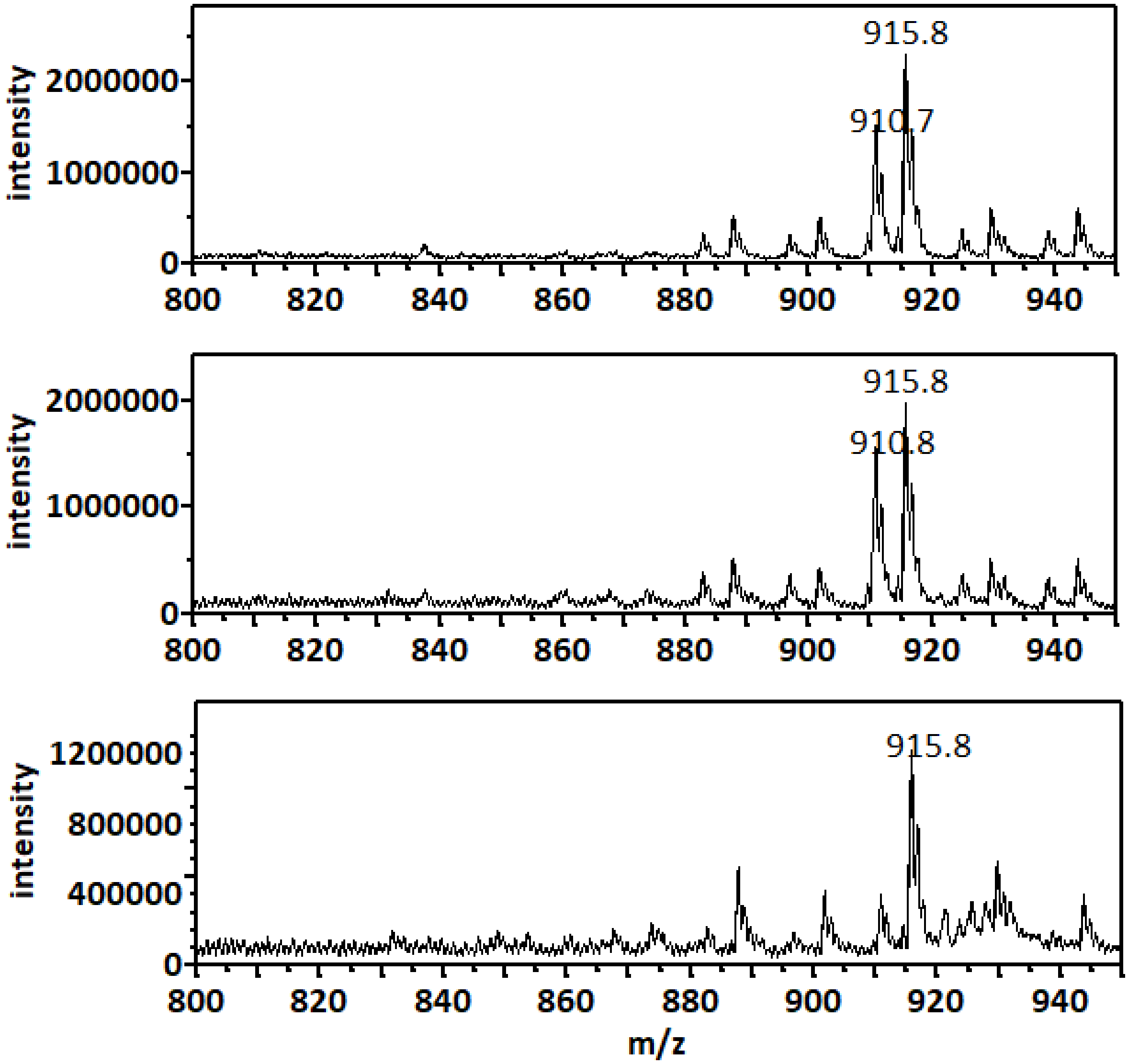

Figure S17. Positive MS spectra of 3 lipid samples of *S. epidermidis* in stationary phase.

In comparison with MS spectra of lipids from S. epidermidis in exponential phase, two clusters of DGDG ions centered around 910 (ammoniated) and 915 (sodiated) m/z were noticeably more abundant. On the other hand, protonated Lysyl-PG ions were absent. We also confirmed that deprotonated lysyl-PG ions were also absent using a precursor scan for -145 m/z lysine anion.

Table S18. Microbial typing results for identifying the *S. haemolyticus* strain.

Test	***Result***
Colonies	White colour
Hemolysis	Positive
Gram stain	Gram +ve, cocci in groups
Catalase test	Positive
Oxidase test	Negative
Mannitol salt agar	Growth +, No color change
6.5% NaCl broth	No growth
Urease expression	Negative
Indole	Negative
Mannitol	Negative
Glucose	Positive
Maltose	Positive
Xylose	Negative
Ribose	Negative
Rhamnose	Negative
Sucrose	Positive
Sorbitol	Negative
Mannose	Negative
Novobiocin Resistance	Negative (sensitive)

The accidentally isolated bacterial strain, was observed to be resistant to at least two common antibiotics ampicillin and erythromycin. Two selective bacterial cultures, with ampicillin and erythromycin respectively, were subjected to microbial typing experiments. The duplicated test results summarised in Table S10, collectively suggest that the strain is likely *S. haemolyticus*. This identification was also supported by MALDI-TOF MS-based clinical microbial typing experiment.

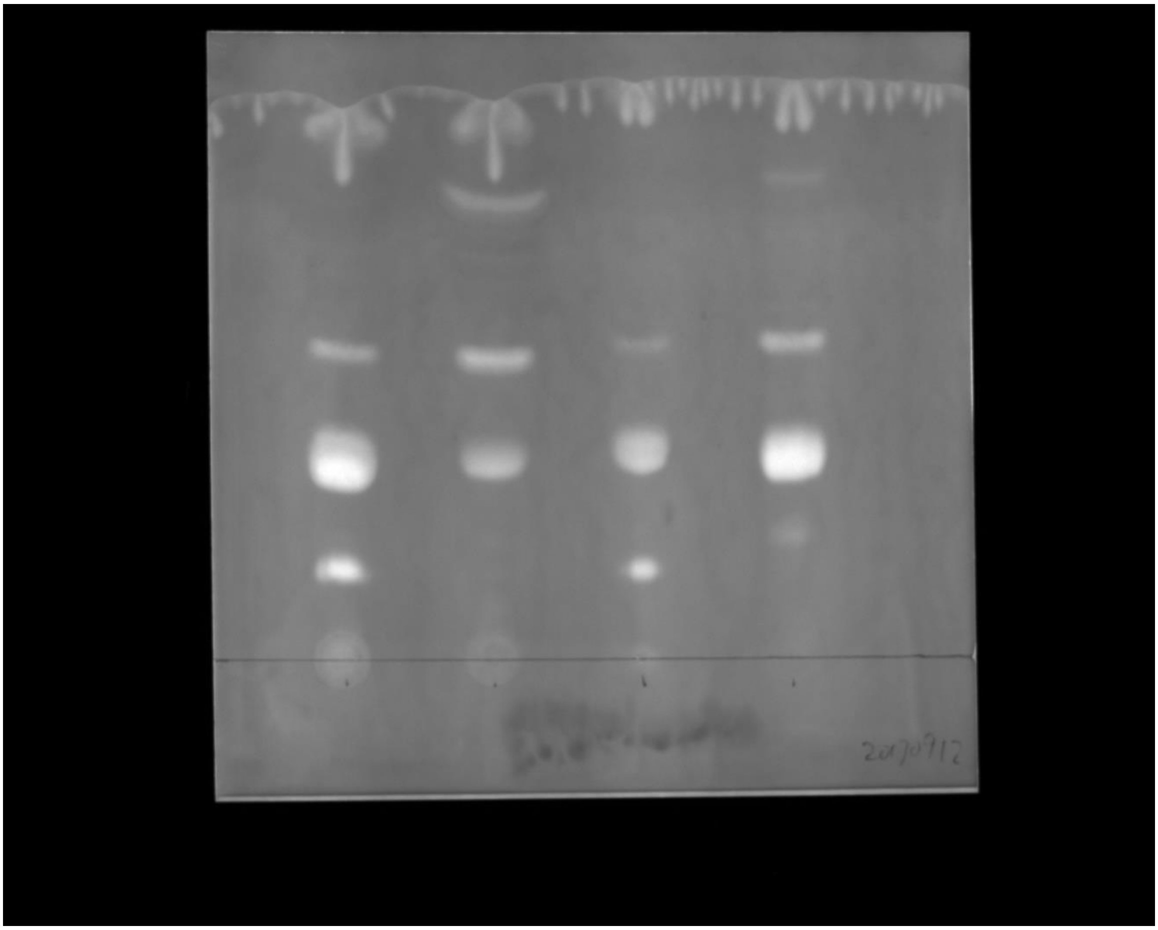

Original chromatogram of Figure 1.

The chromatogram shown in Figure 1 was cropped from this original chromatogram. The TLC sheet was transilluminated by medium wavelength UV. The fluorescence was filtered by a UV filter. No adjustment of brightness or contrast was done to the image. The default settings of brightness and contrast were used. In Figure 1, Hight-width ratio was increased to fit in the overall figure.

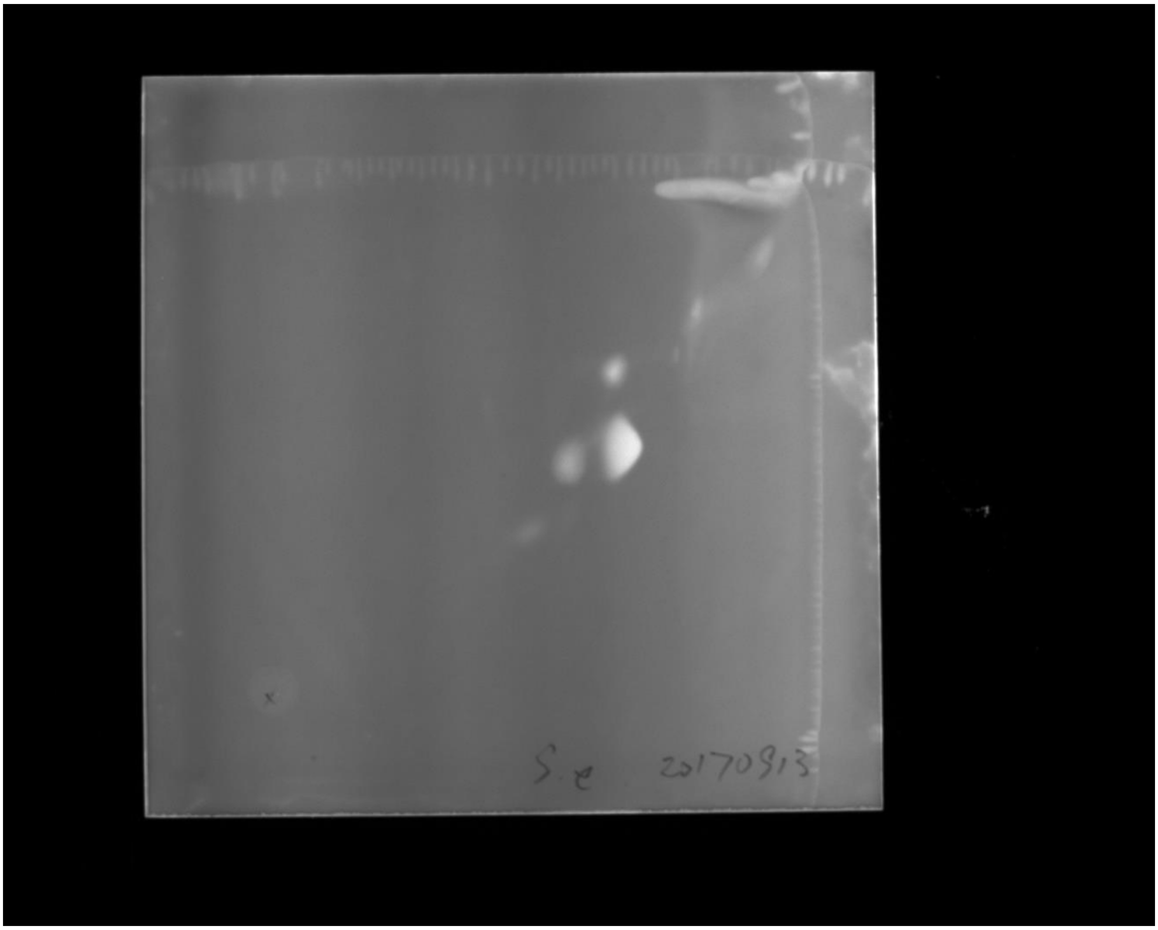

Original chromatogram of Figure 5.

The 2D-TLC chromatogram shown in Figure 5 was cropped from this original chromatogram. The TLC sheet was transilluminated by medium wavelength UV. The fluorescence was filtered by a UV filter. No adjustment of brightness or contrast was done to the image. The full-length chromatogram and default settings of brightness and contrast were used.
